# The carbon quantum dots: Preparation, antibacterial mechanisms, and application in food packaging

**DOI:** 10.1016/j.crfs.2026.101316

**Published:** 2026-01-20

**Authors:** Hongkun Xue, Jiacheng Yang, Hao Yu, Rong Dong, Xiaojun Liao, Jiaqi Tan

**Affiliations:** a College of Traditional Chinese Medicine, Hebei University, No. 342 Yuhua East Road, Lianchi District, Baoding, 071002, China; b School of Clinical Medicine, Hebei University, No. 342 Yuhua East Road, Lianchi District, Baoding, 071002, China; c College of Food Science and Nutritional Engineering, China Agricultural University, No. 17 Qinghua East Road, Haidian District, Beijing, 100083, China

**Keywords:** CQDs, Preparation, Antibacterial activity, Food packaging

## Abstract

In recent years, food spoilage and deterioration caused by microbial contamination have become increasingly serious. Traditional food packaging materials can inhibit the growth and reproduction of microorganisms, thereby extending the shelf life of food. Unfortunately, traditional packaging materials have limitations, including limited antibacterial performance, easy environmental pollution, and poor biocompatibility. Hence, the development of food packaging materials with antibacterial properties has become a research hotspot. Carbon quantum dots (CQDs), as emerging food packaging materials, have excellent antibacterial activity, good biocompatibility, and low toxicity. Therefore, CQDs show broad application prospects in the field of food packaging. This paper initially reviews the preparation methods (arc discharge, laser etching, electrochemical exfoliation, etc.) of CQDs and the selection of carbon sources (carbon-based materials, small molecule organic compounds, and biomass carbon sources). Subsequently, this review systematically summarizes the antibacterial mechanisms of CQDs. Finally, this article comprehensively outlines the application of CQDs in the packaging of fruits and vegetables, meats, seafood, grains, and dairy products. The findings provide theoretical support for the further application of CQDs in the field of food packaging.

## Highlights

• The preparation method of carbon quantum dots are systematically summarized.
• The antibacterial mechanisms of carbon quantum dots are outlined.
• The application in food packaging field of carbon quantum dots is elucidated.

## Graphical abstract

**Figure:**
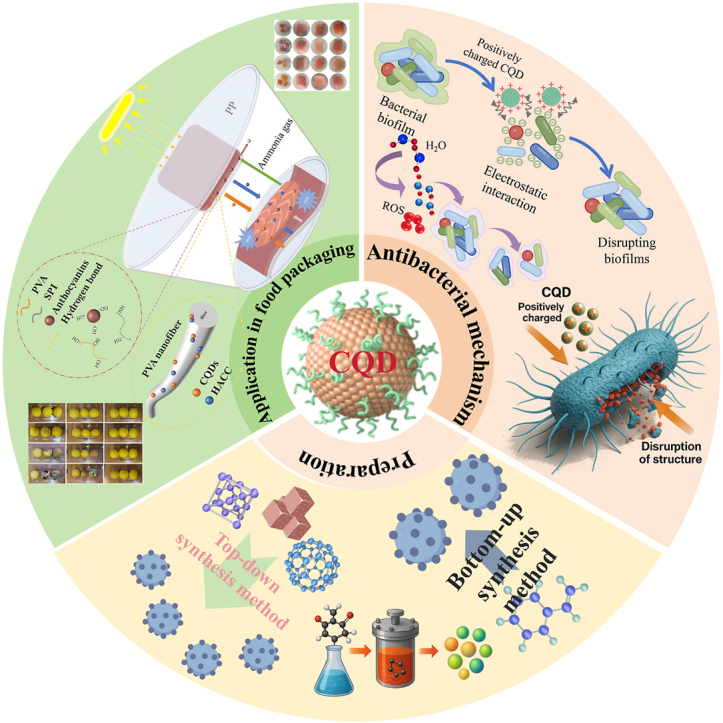

## 1 Introduction

Ensuring food safety is one of the major global challenges facing humanity. Numerous countries have invested a great deal of money in producing healthy and safe food for their people (King et al., 2017). However, food often suffers a relatively large proportion of losses due to spoilage, which not only causes huge economic losses, but also has drawn widespread social attention (Branca et al., 2019). Food is prone to spoilage during storage and distribution, which is mainly attributed to the reproduction of microorganisms or biochemical reactions (Ezati et al., 2022). Consequently, how to effectively inhibit the reproduction and growth of microorganisms on the surface of food has become a core link and key control point to ensure food safety in the processes of food processing, storage, and transportation. Food packaging technology, as one of the techniques to ensure food safety and extend the shelf life of food, is dedicated to preventing food spoilage and reducing losses after food processing (Priyadarshi et al., 2022). Currently, previous report mainly used food packaging materials with antibacterial function, which could effectively inhibit the growth and reproduction of various harmful microorganisms and reduce their metabolic activity and overall quantity. This was conducive to blocking the erosion of food by microorganisms from the source and achieving a slowdown in the rate of food spoilage and deterioration (Dey et al., 2025). The realization of the antibacterial function of packaging materials mainly relies on the active addition of functional components with antibacterial activity in the packaging materials. These active substances could also significantly enhance the comprehensive performance of packaging materials (Ezati and Rhim, 2021; Ghorbani et al., 2021). Nevertheless, common antibacterial materials have limitations, such as a limited antibacterial spectrum, insufficient durability, a single function, and easy production drug resistance. Hence, the development of active antibacterial ingredients with excellent functionality and safety is the core element for the successful research and development of antibacterial food packaging materials.

With the rapid development of nanotechnology, carbon quantum dots (CQDs), as the emerging carbon-based nanomaterials, have shown great application potential in many fields due to their unique optical, electrical, and biocompatibility properties (Lin et al., 2018). CQDs with a size of less than 10 nm are spherical particles, which can exert antibacterial effects through various mechanisms, including damage to bacterial cell walls, destruction of infectious biofilms, and promotion the generation of ROS (Wu et al., 2021a, Wu et al., 2021b; Liu et al., 2020a, Liu et al., 2020b). CQDs are regarded as a strong competitor in the field of food packaging, which is attributed to their sustainability, low cytotoxicity, and excellent biocompatibility (Priyadarshi et al., 2024). In addition, the uniform dispersion of CQDs in the matrix of packaging materials can significantly enhance the mechanical strength of the packaging materials, which is conducive to protecting the structural integrity of the packaging films (Ezati et al., 2022). Notably, CQDs exhibit excellent UV shielding ability due to their unique nanostructures and optical properties. Moreover, the compact surface morphology of CQDs endows them with excellent gas barrier properties, and CQDs have a dual protective mechanisms, which can effectively inhibit the spooling reaction of food during storage, slow down the degradation of nutrients in food and the deterioration of sensory quality, and finally achieve the goal of extending the shelf life of food (Khoshkalampour et al., 2024; Tripathi et al., 2024). Hence, the introduction of CQDs into packaging materials can give packaging materials good antibacterial properties and enhance physical protection function, further implying that CQDs show significant application potential in the field of food packaging. Besides, CQDs nanomaterials have also received extensive attention in the innovative applications of food packaging.

Presently, the existing reviews have insufficient depth and universality in the research on the antibacterial mechanisms of CQDs, and the review on the integration and application efficacy of CQDs in food packaging substrates is limited. To fill these gaps, this review outlines the preparation methods (arc discharge, laser etching, electrochemical exfoliation, etc.) of CQDs and the selection of carbon sources (carbon-based materials, small molecule organic compounds, and biomass carbon sources). Moreover, this paper comprehensively reviews the antibacterial mechanisms of CQDs. Finally, this article systematically summarizes the application of CQDs in the packaging of fruits and vegetables, meats, seafood, grains, and dairy products. The findings can provide important guidance for the industrial application of CQDs in the field of food packaging.

## 2 Preparation methods

**Fig. 1:**
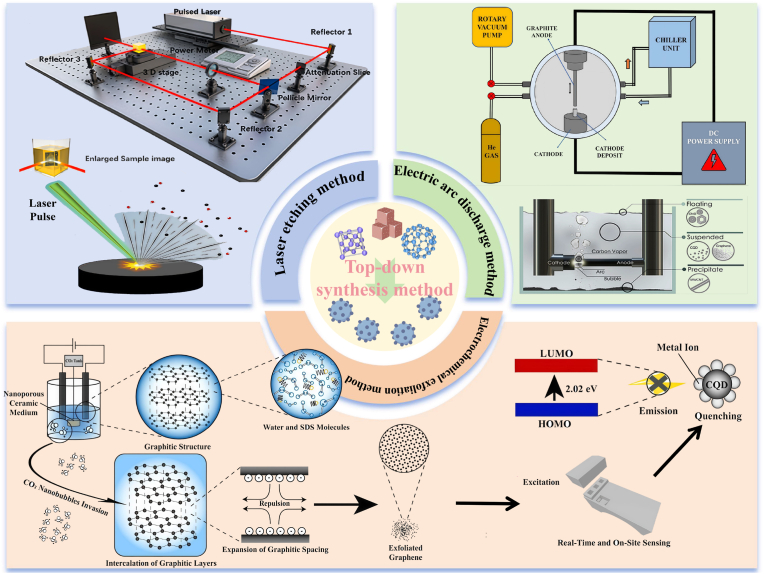
Common top-down methods for synthesizing CQDs (Madhurima et al., 2024; Cui et al., 2020; Calabro et al., 2018; Yat et al., 2024).

**Fig. 2:**
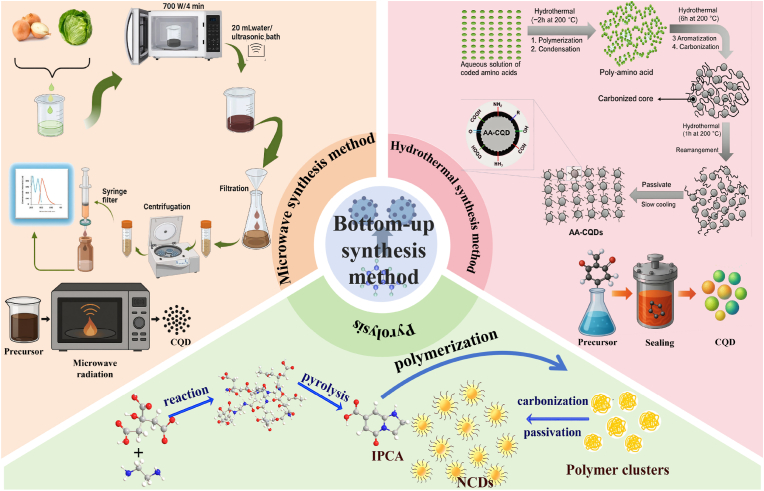
Common bottom-up synthesis methods for CQDs (Kumar et al., 2024; Qandeel et al., 2023; Lai et al., 2021).

CQDs, as the emerging class of low-dimensional nano-materials, have attracted much attention due to their unique functional characteristics (Zare et al., 2025). With the deepening of research and the continuous progress of technology, the preparation methods of CQDs are increasingly diversified. Currently, the preparation of CQDs can be mainly divided into two categories, including “top-down” (Fig. 1) and “bottom-up” (Fig. 2) (Pillar-Little et al., 2018). Large-sized carbon sources are stripped into tiny CQDs by the physical or chemical methods during the “top-down” process. This process typically involves various techniques, such as laser ablation and electrochemical exfoliation, which synthesize CQDs by decomposing carbon-rich substances (Unnikrishnan et al., 2024). The advantages of the “top-down” method are low raw material costs and wide sources. However, this method has limitations such as a complex process, harsh experimental conditions, easy environmental pollution during dissolution, difficulty in precisely controlling the size and fluorescence performance of the product, and low luminescence efficiency. The “bottom-up” approach usually utilizes organic small molecules or oligomers as carbon sources and relies on controllable synthesis and carbonization technologies to achieve the directional preparation of biomass-based CQDs (S. Yang et al., 2023a, Yang et al., 2023b). The “bottom-up” method has some advantages, including mild reaction conditions and simple operation. In addition, this method can precisely regulate the size, surface functional groups, and luminescence performance of CQDs through precursor selection and reaction parameters (Xiong et al., 2021). Unfortunately, the “bottom-up” method has certain drawbacks, such as high raw material costs, toxic precursors, and low product purity. Overall, the “bottom-up” method has some advantages in performance controllability and uniformity, which is suitable for high-performance applications. In contrast, the “top-down” method is more conducive to low-cost and large-scale production. However, the uniformity of product performance is relatively poor. Therefore, choosing the method for synthesizing CQDs requires balancing application requirements, costs, and process conditions. Table 1 presents the preparation methods and reaction conditions of various common CQDs.

**Table 1:** The raw material, preparation method, reaction condition, and application of CQDs.

Raw material	Synthetic method	Response condition	Application	References
Isopropyl alcohol	Hydrothermal method	Heating at 180 °C for 1 h	Fluorescent probe	Xiong et al. (2021)
Grass	Hydrothermal method	Heating at 180 °C for 2 h	Photocatalysis	Sabet & Mahdavi (2019)
Chicken bones	Hydrothermal method	Heating at 180 °C for 4 h	Fluorescent probe	Ye et al. (2022)
Citric acid and urea	Hydrothermal method	Heating at 160 °C for 4 h	Photoelectrocatalysis	Vercelli et al. (2021)
Maple leaves	Hydrothermal method	Heating at 190 °C for 8 h	Biological imaging	Chellasamy et al. (2022)
Urea and o-phenylenediamine	Hydrothermal method	Heating at 180 °C for 12 h	Fluorescent probe	Duan et al. (2018)
Succinic acid and tris (2-aminoethyl) amine	Microwave-assisted method	250 W heating for 5 min	Biological imaging	Chae et al. (2017)
Urea and citric acid	Microwave-assisted method	900 W heating for 4 min	Photocatalysis	Mmelesi et al. (2025)
Xylan	Microwave-assisted method	200 W heating for 10 min	Fluorescent probe	Yang et al. (2018)
Lysine	Microwave-assisted method	750 W heating for 4–6 min	Biological imaging	Choi et al. (2017)
Sugarcane bagasse	Microwave-assisted method	950 W heating for 1 min	Photocatalysis	Kumalayanti et al. (2025)
Graphite rod	Electrochemical exfoliation method	15 V electrolysis for 6 h	Fluorescent probe	Xu et al., 2013
L-lysine	Pyrolysis method	240 °C heating for 3 h	Antimicrobial infection	Xu et al. (2024)
Glucosamine and urea	Pyrolysis method	180 °C heating for 5 min	Fluorescent probe	Li et al., 2022a, Li et al., 2022b, Li et al., 2022c
Orange juice	Hydrothermal method	180 °C heating for 90 min	Fluorescent probe	Gholipour & Rahmani (2024)
Citric acid and glucose	Hydrothermal method	170 °C heating for 12 h	Photocatalysis	Shen et al. (2018)
Citric acid	Hydrothermal method	200 °C heating for 4 h	Fluorescent probe	Wang et al. (2019a)

### 2.1 Top-down method

The top-down synthesis method involves stripping nanoscale carbon particles from large-sized carbon-based frameworks through physical or chemical methods to prepare CQDs (Unnikrishnan et al., 2024). This method relies on strong external forces, such as high temperature, oxidants, or electric arcs, to destroy organic structures, thereby causing carbon chain breakage and decomposition, and forming CQDs (Zare et al., 2025). CQDs have some advantages, including small size, good dispersibility, and good biocompatibility. However, the top-down synthesis method also has limitations, such as a complex preparation process, high cost, and harmful by-products (Rasal et al., 2021). Currently, increasing studies are focusing on the preparation of CQDs by the top-down synthesis method, which can effectively enhance their performance and expand their applications in various fields.

#### 2.1.1 Arc discharge method

The arc discharge method of CQDs, as a typical bottom-up synthesis method, has been widely applied in the preparation of carbon nanomaterials. Arc discharge technology can generate a high-temperature plasma environment through the action of high-energy arcs on graphite electrodes, thereby achieving the evaporation of graphite and the re-condensation of CQDs (Wang et al., 2019c). These carbon vapors rapidly condense during the cooling process, thereby forming carbon nanoparticles or CQDs (Sharma and Soni, 2024). Presently, numerous studies have focused on CQDs by the arc discharge method. For example, Xu et al. (2004) prepared the single-walled carbon nanotubes (SWCNs) by the arc discharge method, and fluorescent carbon nanoparticles were isolated from SWCNs for the first time, further implying that the arc discharge method can be adopted as a novel and effective approach for electrophoretic analysis and purification of fluorescent single-walled carbon nanotube fragments, which provides important technical support for research related to carbon nanotubes. Dey et al. (2014) prepared CQDs doped with boron and nitrogen elements that emitted intense blue light by the arc discharge method. The results show that CQDs had PL emission peaks independent of the excitation wavelength. In addition, the PL emission peaks of CQDs doped with different elements were concentrated between 400 nm and 425 nm. Among them, the emission peak of B-CQDs was the longest, which was mainly attributed to the existence of π-π bonds and was conducive to the generation of trap states in CQDs, thereby causing differences. Interestingly, CQDs prepared by arc discharge technology have good fluorescence. However, the yield of CQDs is low, and the particle size is not uniform, which is not suitable for industrial production. Accordingly, some researchers have modified the traditional arc discharge technology to improve the yield and performance of CQDs. For example, carbon nanomaterials were prepared by using the improved arc discharge technology under specific electrode materials and reaction conditions (Madhurima et al., 2024). The results show that carbon nanomaterials had a unique microstructure, and their size and shape met the expectations. Moreover, carbon nanomaterials possessed specific crystal structures and lattice vibration modes, which laid the foundation for clarifying their adsorption performance. In another study, CQDs were prepared by the underwater arc discharge method and explored the influence of different conditions on the synthesis of CQDs (Chao-Mujica et al., 2021). The results show that CQDs with a size distribution within the range of 1–5 nm were ultimately obtained by adjusting parameters, including voltage, current, and discharge time, and their fluorescence quantum yield was 16 %. Moreover, CQDs had high crystal quality, good photoluminescence performance, and high tolerance to salt and metal ions. Furthermore, CQDs showed high selectivity and sensitivity to tetracycline antibiotics, implying that the arc discharge method is a green, economical, and fast method to synthesize CQDs. Unfortunately, the high temperature and high energy input during the arc discharge process may introduce impurities, which in turn affect the performance and stability of CQDs and limit their application in the field of materials science (Kong et al., 2024).

In summary, CQDs with specific fluorescence characteristics can be obtained by the arc discharge method. The arc discharge method provides a potential strategy for the synthesis of CQDs with high crystallicity and in situ doping. Moreover, the arc discharge method has some advantages, including simple operation, environmental friendliness, and a large amount of preparation. However, the traditional arc discharge method has some defects, such as low yield and uneven particle size distribution, which limit its application in industrial production. Existing studies can improve material properties and quantum yield by enhancing the traditional arc discharge technology. In the future, the pulsed arc technology, supercritical fluid-assisted discharge method, and multi-physics coupling control system can be used to prepare CQDs with high performance and high stability.

#### 2.1.2 Laser etching method

Laser etching technology is a method for preparing CQDs by irradiating carbon-containing precursors with a laser beam to exfoliate and purify carbon nanoparticles (Kong et al., 2024). The principle of this method is to irradiate the carbon source with laser pulses to create a high-temperature and high-pressure environment, which in turn promotes melting, vaporization, and recrystallization on the surface of the carbon source and forms CQDs with fluorescent properties (Reyes et al., 2016; Qureshi et al., 2024). Sun et al. (2006) prepared CQDs by the laser etching method. The results show that CQDs had excellent photoluminescence properties and stability. In addition, CQDs exhibited low cytotoxicity and good biocompatibility, further suggesting that CQDs show great application potential in the field of biological imaging. Unfortunately, the preparation of CQDs by laser etching technology has limitations, such as low yield, a complex preparation process, and high cost. Hence, increasing studies are dedicated to improving the laser etching method to enhance the yield and performance of CQDs. Cui et al. (2020) prepared CQDs by using a self-made dual-beam pulsed laser etching system. The results exhibit that this method could shorten the laser ablation time, while increasing the quantum yield of CQDs (35.4 %). Moreover, CQDs had a uniform particle size distribution, which was attributed to the fact that the dual-beam pulsed laser etching system could precisely control the energy distribution, thereby achieving controllable synthesis of the CQDs size. Furthermore, CQDs showed good stability and luminescence characteristics. Similarly, Nguyen et al. (2020) prepared CQDs via the dual-pulse femtosecond laser etching technology. The results show that the CQDs prepared by this technology had a small particle size and a high specific area, which was conducive to improving their surface activity and functionalized modification ability. Besides, the second pulse promoted the generation of rich functional groups on the surface of CQDs by reheating the newly ablated material, thereby enhancing the application of CQDs in the fields of sensing and catalysis. With the improvement of science and technology, liquid phase laser etching is also used as a promising preparation technology for CQDs. Compared with the chemical method, CQDs prepared by liquid-phase laser etching were smaller in size, faster in speed, and more environmentally friendly. In addition, the precursors of CQDs were inexpensive and widely available, which was conducive to further reducing production costs and achieving large-scale preparation (Calabro et al., 2018).

In summary, CQDs produced by laser etching have some advantages, including stable chemical properties, relatively high yield, and short production time. Nevertheless, the laser etching method also has some limitations, such as complex operation and high cost. At present, the laser etching method has been continuously improved. Unfortunately, the laser etching method also faces certain challenges as follows: 1) How to accurately control the crystallinity of CQDs; 2) How to improve the product purity of CQDs; 3) How to reduce production cost and simplify the operation of CQDs. Future research should focus on addressing the limitations of laser etching method through the following approaches: 1) We can use advanced optimization algorithms to optimize quenching conditions and annealing processes, thereby achieving precise control of the crystallinity of CQDs; 2) We can utilize high-purity target materials and background media to optimize the design of the reaction chamber, thereby enhancing the purity of CQDs and expanding their application range; 3) We can develop efficient and stable continuous flow reaction systems, optimize the utilization rate of target materials, enhance the efficiency of laser energy conversion, thereby preparing high-performance, low-cost, and excellent functional properties of CQDs.

#### 2.1.3 Electrochemical exfoliation method

Electrochemical exfoliation technology is a strategy that uses carbon materials (graphite) as electrodes and promotes REDOX reactions by applying specific voltages or currents, thereby exfoliating the carbon materials into CQDs (Miao et al., 2017; Miao et al., 2017a, Miao et al., 2017b; Liu et al., 2016). Electrochemical exfoliation technology has some advantages, including simple operation, abundant raw materials, and low cost. In addition, CQDs prepared by the electrochemical exfoliation method show high yield, complete crystallization, and are easy to purify (Xia et al., 2025b). Currently, growing studies have prepared CQDs with excellent performance by the electrochemical exfoliation method. For example, Zhao et al. (2023) prepared AL-CQDs by using electrochemical exfoliation technology. The results show that the average particle size of Al-CQDs was 2.7 nm, and Al-CQDs had excellent ultraviolet absorption and fluorescence emission capabilities. Besides, Al-CQDs showed a significant ROS clearance ability (the clearance rate of 72.37 %), which was mainly attributed to the good charge transfer ability of Al-CQDs. In another study, CQDs were prepared via the electrochemical exfoliation method using an inorganic solution and a graphite rod as the electrolyte and electrode, respectively. The results show that CQDs had good photoluminescence performance, which was mainly attributed to the quantum confinement effect of the carbon core of CQDs. Moreover, this preparation method exhibited low process cost, simple operation, and green pollution-free, which was conducive to the large-scale production of CQDs (Tian et al., 2020). Yu et al. (2024) synthesized CQDs by the electrochemical exfoliation method and then prepared flexible electronic synapses using CQDs. The results show that the flexible electronic synapses could still maintain a similar function to their initial state after multiple bends. This was because the CQDs synthesized by the electrochemical exfoliation method had few surface defects, which was conducive to the flexible electronic synapses demonstrating good density and uniformity. Additionally, the electrochemical exfoliation method demonstrated some advantages, such as good controllability, ease of operation, and low cost, implying that the electrochemical exfoliation method is an ideal choice for preparing high-performance CQDs. Furthermore, CQDs exhibited excellent electrochemical stability and conductivity, further suggesting that CQDs synthesized by the electrochemical exfoliation method can provide a reliable material basis for device development. Unfortunately, the electrochemical exfoliation method is prone to corrosion by strong acid/strong alkali electrolyte solutions and relies on high-purity graphite electrodes. To address the above limitations, numerous studies are focusing on enhancing electrolyte solutions and improving the electrochemical exfoliation method, thereby reducing production cost and increasing production efficiency of CQDs. Li et al., 2024a, Li et al., 2024b prepared CQDs with excellent performance via the electrochemical exfoliation method using hydrogen peroxide solution as the electrolyte. Compared with the traditional electrochemical exfoliation method, the yield and carboxyl content of CQDs prepared by the improved electrochemical exfoliation method had increased by 1.49 times and 2.01 times, respectively. In addition, CQDs showed excellent buffering capacity. It was mainly attributed to the abundant carboxyl functional groups on their surface that could dissociate or combine with protons, thereby effectively regulating the acid-base balance of the surrounding environment. Furthermore, CQDs had been widely applied in phenol degradation. In another study, CQDs were prepared by combining the electrochemical exfoliation method with carbon dioxide nanobubble technology. The results show that the quantum yield and average particle size of CQDs were 26.17 % and 2.24 ± 1.07 nm, respectively. Moreover, the composite of carbon dioxide nanobubbles increased the carbon exfoliation rate, which was conducive to the large-scale production of CQDs. Furthermore, CQDs had good selectivity for Fe^3+^, further indicating that CQDs also show great application potential in the field of heavy metal detection (Yat et al., 2024).

In summary, the electrochemical exfoliation method has some advantages, such as simple operation, high output, and low production cost. Additionally, the electrochemical exfoliation method can enhance the performance and application scope of CQDs through specific improvements. However, the electrochemical exfoliation method still has the following limitations in practical applications: 1) The process of preparing CQDs by electrochemical exfoliation is affected by multiple factors, which is not conducive to achieving completely synchronous and uniform preparation of high-performance CQDs; 2) The exfoliation process of the electrochemical exfoliation method is sensitive to reaction parameters, and minor changes in reaction conditions can lead to significant differences in the performance of CQDs and poor repeatability; 3) The intense electrochemical oxidation process is prone to introduce sp^3+^ defects or vacancies in the carbon nucleus, thereby destroying the sp^2+^ conjugated structure and reducing the luminescence efficiency. To the above limitations, future research should focus on the following aspects to optimize the electrochemical stripping method: 1) We can optimize reaction parameters, design three-dimensional porous electrodes, control intercalation and exfoliation rates, and improve the uniformity of the exfoliation process.; 2) We can adopt automated equipment to control the reaction process, and combine online spectroscopy, electrochemical monitoring, and other means to provide real-time feedback and adjust parameters for ensuring the reproducibility between batches; 3) We can adopt cathodic exfoliation/bipolar electrochemical strategies to reduce oxidative damage and enhance the performance of CQDs. In the future, we can continuously modify the electrochemical exfoliation method through technological innovation, which is expected to become an effective strategy for preparing efficient and stable CQDs.

### 2.2 Bottom-up method

The “bottom-up” method typically uses organic small molecules or oligomers as precursors and carbonizes the precursors under certain conditions, thus forming CQDs. The “bottom-up” method mainly includes hydrothermal/solvothermal, microwave synthesis, pyrolysis, and other methods (Lai et al., 2021). The crystallinity and structural integrity of CQDs prepared by the “bottom-up” method are relatively poor. Interestingly, the precursors for the “bottom-up” synthesis of CQDs are very extensive. The “bottom-up” method can precisely control the size, shape, and fluorescence properties of CQDs by designing the structure, reaction time, solvent, and temperature of the reaction precursors, which provides the possibility for the large-scale production and application of CQDs (Yadav et al., 2023).

#### 2.2.1 Hydrothermal/solvothermal method

The hydrothermal/solvothermal method is a “bottom-up” synthesis strategy based on liquid-phase reactions. This method can utilize high-temperature and high-pressure reaction conditions to promote the carbonization and self-assembly of organic precursors to form CQDs (Kong et al., 2024; Tajik et al., 2020). The hydrothermal/solvothermal method has some advantages, including simple operation, controllable reaction conditions, low cost, and environmental friendliness. Therefore, the hydrothermal/solvothermal method is widely applied in the synthesis of CQDs (Banger et al., 2023). Presently, increasing studies are focusing on preparing CQDs with excellent performance through the hydrothermal/solvothermal method. Thakur et al. (2024) synthesized PP-CQDs by the hydrothermal method using papaya peel (PP) as the raw material. The results exhibit that the average particle size and quantum yield of PP-CQDs were 4.16 nm ± 0.07 nm and 39.15 %, respectively. In addition, PP-CQDs had excellent stability, good fluorescence characteristics, a wide absorption spectrum, and significant antibacterial properties. Furthermore, PP-CQDs nano-hydrogel complex could significantly reduce the weight loss of strawberries, maintain their original physicochemical properties, and inhibit the growth of various microorganisms. In another study, CQDs nano-fluorescent probes were obtained by the hydrothermal method using corn stalks as raw materials and evaluated their detection effect on methyl parathion. The results exhibit that the particle size distribution and dispersion of CQDs were uniform and good. Moreover, the hydrothermal method could achieve the green recycling of biological waste, which provided new ideas for the sustainable preparation and application of CQDs. Furthermore, CQDs exhibited excellent sensitivity and accuracy for methyl parathion (detection limit 1.22 μg/kg and quantification limit 4.07 μg/kg), further suggesting that CQDs show great potential in the field of sensing (Zhang et al., 2023). Chen et al. (2020) synthesized tellurium-doped CQDs (Te-CQDs) via the hydrothermal method using cysteine tellurium as the precursor. The results display that Te-CQDs exhibited good application prospects in the biomedical field, which was attributed to their excellent optical performance and good biocompatibility. Additionally, Te-CQDs could eliminate H_2_O_2_ and generate ROS under 808 nm laser irradiation and effectively promote photodynamic therapy, which provided a new idea and strategy for cancer treatment. Unfortunately, the traditional hydrothermal/solvothermal method also has limitations, such as long reaction time, a large number of by-products, and low yield. To address the limitations of the traditional hydrothermal method, Jing et al. (2019) improved the traditional hydrothermal method for preparing CQDs by using mild oxidation pretreatment. The results show that the improved traditional hydrothermal method could change the structure and composition of biomass-derived carbon, reduce the generation of by-products, and improve the synthesis efficiency and yield of CQDs (76.90 wt%), further suggesting that the improved hydrothermal method can provide a novel, simple, and effective approach for large-scale synthesis of CQDs. Moreover, the improved hydrothermal production process showed significant environmental friendliness and economic advantages. Similarly, Liu et al. (2025) synthesized N-CQDs via the hydrothermal method using citric acid as the carbon source and ethylenediamine monohydrate as the nitrogen source. The results show that N-CQDs exhibited a unique “biphasic luminescence behavior” with the variation of N content. The dehydration rate of citric acid accelerated with the increase of the doping amount of N element, which was conducive to N-CQDs having a high luminescence quantum yield and photoluminescence lifetime. In another study, CQDs with superior performance were prepared by using 20 encoded amino acids as precursors through four stages of dehydration, polymerization, protection, and carbonization by an improved hydrothermal method. The results exhibit that the diameter range of CQDs was from 3.0 nm ± 0.5 nm–9.0 nm ± 0.8 nm, and the quantum yield of CQDs was 86 %. Moreover, CQDs showed good biocompatibility and low cytotoxicity, implying that CQDs have promising application prospects in fields such as bioimaging and biosensing (Kumar et al., 2024).

In summary, the hydrothermal/solvothermal method serves as an efficient “bottom-up” strategy for synthesizing CQDs. This method has some advantages, including simple operation, high yield, and precise regulation of CQDs by changing the types of precursors and reaction parameters, which provides a solid foundation for the wide application of CQDs. However, the hydrothermal/solvothermal method still faces certain limitations as follows: 1) The “black box” properties of the hydrothermal process leads to unclear intermediate products and bonding mechanisms, which is not conducive to the directional design of CQDs; 2) The performance of CQDs prepared by hydrothermal/solvothermal synthesis is easily affected by carbon sources and solvents, thus showing poor repeatability; 3) The hydrothermal method may destroy the active groups of CQDs, which limits their application in various fields. Hence, the hydrothermal/solvothermal method should focus on making breakthroughs in the following aspects: 1) We can further reveal the carbonization and polycondensation pathways by combining in-situ characterization and theoretical calculation to achieve efficient preparation of CQDs; 2) We can develop standardized precursor combinations or adopt microwave-assisted hydrothermal method to further enhance the consistency of CQDs; 3) We can adopt stepwise synthesis and “one-pot method” co-reaction to enhance the physicochemical properties and functional activities of CQDs. In the future, we can optimize the synthesis process through the integration of multiple disciplines, which is expected to achieve efficient and directional preparation of CQDs.

#### 2.2.2 Microwave-assisted synthesis method

Microwave-assisted synthesis is a technique that uses microwave radiation heating to drive carbon sources for carbonization reactions and prepares CQDs by controlling reaction conditions such as microwave radiation power and temperature (Kong et al., 2024). Compared with the traditional heating method, microwave radiation heating has a shorter reaction time and a higher energy utilization rate. This is mainly attributed to the fact that microwaves can rapidly penetrate the reaction system, which is conducive to the reactants reaching the reaction temperature in a short time, thereby promoting the rapid formation of CQDs (Tajik et al., 2020; Khayal et al., 2021). Currently, increasing studies are focusing on the preparation of CQDs by the microwave-assisted synthesis method. For example, Jeong et al. (2021) prepared CQDs with excellent performance by the microwave-assisted synthesis method using EDTA and TTDDA monomers as precursors. The results show that the mass yield and photoluminescence quantum yield of CQDs were 25 wt% and 53.3 %, respectively. In addition, CQDs could dissolve in polar solutions (water, ethanol, and methanol), which was attributed to the presence of polar functional groups (hydroxyl groups, amines, and carboxylic acids) on their surface. Furthermore, CQDs also showed excellent fluorescence properties, low cytotoxicity, and good biocompatibility. In another study, CQDs were prepared via the microwave-assisted synthesis method using citric acid and urea as raw materials. The results exhibit that the quantum yield and Tokes shift of CQDs were 73 % and 0.65 eV, respectively. Moreover, CQDs displayed excellent optical performance and could be used to produce high-level bright fluorescent codes (Li et al., 2024a, Li et al., 2024b). Kumar et al. (2020) prepared hydrophilic CQDs by microwave-assisted synthesis method using an aqueous solution of citric acid and urea as precursors. The results show that the band gap and optical properties of CQDs increased with the increase of sample heating time, which was attributed to the fact that the increase of heating time contributed to increasing the density of states of the sample, thereby enhancing the optical performance of CQDs. Moreover, the heating duration could be changed during the microwave-assisted synthesis of CQDs, and the controllable synthesis of CQDs could be achieved without high temperatures and complex chemical techniques, which was conducive to reducing preparation costs and improving synthesis efficiency. Unfortunately, CQDs prepared by the microwave-assisted synthesis method have limitations, such as high preparation cost and easy environmental pollution. Qandeel et al. (2023) prepared S,N-CQDs by microwave-assisted synthesis method using onion and cabbage juice as raw materials. The results show that the average size and fluorescence quantum yield of S,N-CQDs were 12.6 nm ± 3.16 nm and 15.2 %. Besides, S,N-CQDs exhibited good water solubility and stability. Furthermore, S,N-CQDs had demonstrated excellent sensitivity (LOD of 0.07 μm and LOQ of 0.24 μm), further indicating that S,N-CQDs have great application potential in the field of biosensing. In another study, CQDs were synthesized through the microwave-assisted synthesis method using menthol leaves as raw materials. The results display that CQDs exhibited bright blue fluorescence under UV radiation. The maximum emission in the blue region of CQDs was 436 nm at an excitation wavelength of 340 nm. In addition, CQDs had high biocompatibility, low cytotoxicity, and excellent sensitivity, which could be used to detect the concentration of Fe^3+^ (Architha et al., 2021).

In summary, CQDs prepared by the microwave-assisted synthesis method have significant advantages, such as high efficiency, environmental friendliness, high yield, and low cost, which provide a new research direction for the development of green chemistry and nanomaterial science. However, the microwave-assisted synthesis method still has the following limitations in large-scale practical applications: 1) Local overheating caused by microwave heating can lead to a wide size distribution of CQDs, thereby affecting their fluorescence performance and subsequent applications; 2) The rapidity of microwave heating can make it difficult to precisely control the reaction process, thus affecting the properties of CQDs; 3) Laboratory-scale microwave synthesis is difficult to scale up directly and limited yield. To address the above limitations, future research should focus on optimizing CQDs in different methods as follows: 1) We can adjust the microwave power, reaction time, and precursor concentration, thereby improving the uniformity and functional properties of CQDs; 2) We can adopt the gradient heating method and dynamically adjust the microwave parameters to avoid excessive local carbonization during the reaction process, thus preparing functionally adjustable CQDs; 3) We can utilize continuous flow microwave reactors, achieve continuous production of CQDs, and increase the yield through microfluidic control systems. CQDs prepared by the microwave-assisted synthesis method will demonstrate broad application prospects in fields such as photocatalysis and biosensing in the future.

#### 2.2.3 Pyrolysis method

Pyrolysis is a method that promotes chemical reactions (decomposition and carbonization) of organic carbon sources under high-temperature conditions, thereby generating CQDs (Rasal et al., 2021). Compared with the traditional preparation method, the pyrolysis method shows lower cost and simpler operation process (Wu et al., 2023). In addition, the pyrolysis method can flexibly regulate the size, morphology, surface chemical properties, and luminescence intensity of CQDs by changing the type of carbon source, adjusting the temperature, and controlling the reaction duration, thereby expanding the application range of CQDs (Zhang et al., 2022; Karagianni et al., 2023). Currently, increasing studies are focusing on the preparation of CQDs by the pyrolysis method. Ye et al. (2018) prepared inorganic-organic hybrid CQDs through the pyrolysis method using citric acid as the precursor. The results show that the CQDs had good thermal stability, oxidation resistance, and photoluminescence performance. Moreover, CQDs had excellent anti-wear performance, which was mainly attributed to the fact that CQDs could act as bearing balls, thereby significantly reducing the contact area between the friction materials. In another study, L-lysine was used as the carbon source to prepare PL-CQDs by the pyrolysis method. The average particle size and lattice spacing of PL-CQDs were 2.3 nm ± 0.36 nm and 0.21 nm, respectively. Moreover, PL-CQDs had excellent antibacterial activity and could effectively remove bacterial biofilms. This was attributed to the fact that PL-CQDs possessed an ultra-small size, a high positive surface charge, and the ability to generate ROS (Xu et al., 2024). Currently, increasing studies are focusing on the preparation of CQDs using green materials. Yuncu et al. (2024) used hazelnut shell waste as a precursor to prepare CQDs by the pyrolysis method. The results show that he pyrolysis method could reduce the pollution caused by waste treatment, which was conducive to solving environmental pollution problems. Additionally, the synthesized CQDs exhibited excellent photoluminescence, good stability, and high water solubility. Similarly, Özbek et al. (2024) used green and renewable wolfberry seeds as precursors to prepare CQDs with fluorescent properties by the pyrolysis method. The results exhibit that CQDs emitted intense blue fluorescence under ultraviolet lamp (365 nm), and the fluorescence intensity of CQDs remained stable within the pH range of 4.8–10.8. In addition, CQDs had good selectivity for Hg^2+^. In another study, magnetic fluorescent CQDs (MFCQDs) were prepared via the pyrolysis method by combining waste crab shells with transition metal ions (Gd^3+^, Mn^2+^, and Eu^3+^). The results exhibit that MFCQDs showed excellent biocompatibility and low cytotoxicity, which was conducive to their application in the biomedical field. In addition, MFCQDs possessed excellent magnetic and fluorescence properties, and MFCQDs could be used for multimodal biological imaging and targeted drug delivery. Furthermore, MFCQDs could also precisely control the fluorescence emission wavelength of materials by adjusting the types and proportions of metal ions, thereby meeting the needs of different biological detection scenarios (Yao et al., 2017).

In summary, the pyrolysis method is a key technology for synthesizing CQDs. The pyrolysis method shows unique advantages, such as simple operation, low production cost, and environmental friendliness. Therefore, the pyrolysis method is widely applied in various fields. Unfortunately, the pyrolysis method still has certain limitations in practical applications as follows: 1) Material accumulation is prone to cause uneven internal temperature gradients and reaction atmospheres in larger-scale reactors, which in turn leads to the generation of heterogeneous CQDs; 2) The by-products generated during the pyrolysis reaction are difficult to be completely removed, which affects the purity of CQDs; 3) The high-temperature pyrolysis process involves complex carbonization, polymerization, and cracking reactions, and the reaction pathways are difficult to precisely control. To address the above limitations, future research should focus on optimizing the pyrolysis method as follows: 1) We can develop mobile reactors to enable materials to move within the reactor and reduce the temperature gradient; 2) We can design a more effective purification process to remove uncarbonized small molecules, large carbon particles, and other impurities, thereby improving the purity of CQDs; 3) We can adopt programmable temperature-controlled furnaces or more advanced heating technologies to precisely control the reaction temperature and time, and optimize the carbonization process. In the future, we can promote pyrolysis to become a reliable way to produce high-performance and low-cost CQDs through continuous technological innovation and process improvement, which can further facilitate the application of CQDs in fields such as environmental monitoring, biomedicine, and food safety.

## 3 Carbon source selection for CQDs

The fluorescence characteristics, quantum yield, size distribution, surface chemical state, and electrochemical properties of CQDs depend on the type of carbon source and synthesis pathway to a certain extent (Ateia et al., 2024). Carbon sources provide the basic elements for carbon nucleus formation, and their own chemical structure, functional groups, and impurities also have a profound impact on the nucleation, growth, and surface passivation processes of CQDs (Dhariwal et al., 2024). This section mainly classifies carbon sources into three major categories (carbon-based materials, small-molecule organic compounds, and biomass carbon sources) and systematically discusses their influence on the final performance of CQDs.

### 3.1 Carbon-based materials

During the preparation of CQDs, ready-made carbon-based materials can be used as carbon sources. Macroscopic or micrometer-scale carbon structures can be stripped and broken down into nanoscale CQDs through “top-down” physical or chemical methods. This type of carbon source mainly includes graphene, carbon nanotubes, carbon black, graphite, activated carbon, etc. (Shi et al., 2022). The prepared CQDs usually have a high degree of crystallinity, clear lattice fringes, and a carbon core structure closer to that of graphene sheets, which is attributed to the highly graphitized sp^2^ carbon structure of carbon-based materials (Huang et al., 2014). In addition, when carbon-based materials are used as carbon sources to prepare CQDs, a large number of oxygen-containing functional groups (carboxyl and carbonyl) can be introduced onto the surface of CQDs, which is conducive to the generation of intense fluorescence in CQDs (Borna et al., 2021). Presently, increasing studies are focusing on the preparation of CQDs with fluorescent properties through carbon-based materials. For instance, Tan et al. (2019) prepared CQDs using activated carbon as the raw material by the oxidation method and evaluated their fluorescence characteristics. The results show that the fluorescence of CQDs could be adjusted from yellow to green, which was attributed to the fact that highly graphitized activated carbon provided CQDs with a rich sp^2^ carbon domain and more carboxyl groups. In addition, the highly graphitized structure of activated carbon could increase the size of the CQDs conjugated structure and promote radiation recombination, thereby significantly reducing the fluorescence self-absorption phenomenon. In another study, CQDs were prepared by the solvothermal method using graphite as the carbon source, and their structure and optical properties were evaluated (Liu et al., 2024). The results show that CQDs had a uniform structure, clear lattice fringes, and a layered structure similar to that of graphite. Moreover, functional groups (carboxylic acids and hydroxyl groups) were detected on the surface of CQDs. In addition, the surface electrons of CQDs could be excited by a 405 nm laser, rapidly transition to higher energy levels, and then return to the ground state through radiative transitions, thereby emitting intense blue fluorescence. Similarly, Yat et al. (2024) prepared CQDs using graphite as the carbon source by electrochemical exfoliation. The results show that the synthesized CQDs had an amorphous dual-phase carbon nucleus structure and a uniform particle size distribution, which was conducive to improving the dispersion stability of CQDs. In addition, the CQDs inherited the sp^2^ structure of graphite and could undergo significant π-π electron transitions, thereby demonstrating excellent fluorescence performance and good photostability. Unfortunately, compared with small-molecule organic compounds, the process of preparing CQDs using carbon-based materials usually requires a more corrosive environment, and the controllability and uniformity of the products are insufficient (Wang et al., 2019c). Meanwhile, compared with biomass carbon sources, the surface functional group types of CQDs prepared from carbon-based materials are relatively more single, which greatly limits their application scope in different fields (Wu et al., 2023).

In summary, carbon-based materials, as carbon sources, have advantages such as high purity, clear structure, and the ability to introduce surface functional groups. However, carbon-based materials have limitations, including poor environmental friendliness and a relatively single type of surface functional group. In the future, we should optimize from the following aspects: 1) Develop microwave-assisted, ultrasonic-assisted, and other synthesis processes to improve the utilization rate of raw materials and reduce the generation of waste in batch production; 2) Nitrogen, sulfur, phosphorus, and other atoms are introduced to enrich the types of surface functional groups by using atomic doping technology; 3) Molecular chains with specific functions are grafted to achieve the directional regulation of the surface functions of CQDs by using covalent grafting technology. CQDs using carbon-based material sources as carbon sources will achieve dual breakthroughs in precise atomic structure design and precise functional performance regulation, which is conducive to further expanding their applications in fields such as photoelectrocatalysis, biosensing, and food safety detection in the future.

### 3.2 Small-molecule organic compounds

Small-molecule organic compounds (SMOCs) are the most commonly used carbon sources for the synthesis of CQDs through the “bottom-up” method. SMOCs have advantages such as strong designability, high product purity, and good uniformity. Currently, the carbon sources of SMOCs mainly include monomers or simple polymers such as citric acid, urea, amino acids, glucose, ethylenediamine, and polyethyleneimine (Vercelli et al., 2021). The preparation of CQDs using such carbon sources has a high degree of designability. The core structure and surface chemical properties of CQDs can be designed by precisely selecting different small molecule precursors and regulating their ratios (Nguyen et al., 2024). Notably, the chemical composition, size, and performance uniformity of the prepared CQDs were good, which was attributed to the high purity of SMOCs, and the reaction was usually carried out in a closed environment, which was conducive to the batch preparation and standardized application of CQDs (Wu et al., 2017). In recent years, the preparation of CQDs using SMOCs as carbon sources has become a research hotspot. Zheng et al. (2020) synthesized CQDs by the solvothermal method using 2, 7-dihydroxynaphthalene as the carbon source and ethylenediamine as the nitrogen source, and evaluated the effects of different parameters on the performance of CQDs. The results show that the fluorescence quantum yield of CQDs could be effectively regulated by adjusting reaction parameters such as the addition amount of ethylenediamine, reaction temperature, and reaction time. When the addition amount of ethylenediamine, reaction temperature, and reaction time were 4 mL, 180 °C, and 12 h, respectively, the fluorescence quantum yield of the prepared CQDs reached the maximum value (62.98 %). In addition, the synthesized CQDs with an average particle size of 3.31 nm were spherical in shape and emitted bright green fluorescence under ultraviolet light. In another study, CQDs were prepared using citric acid as the carbon source, and the effects of different nitrogen dopants on the structure and properties of CQDs were explored (Nguyen et al., 2024). The urea and trimethylamine groups contributed to the formation of high quantum yields and uniform CQDs, which was attributed to the fact that urea and trimethylamine groups could promote the decarboxylation and dehydration of citric acid, thereby facilitating the formation of graphite carbon nuclei. In addition, amino acids can increase the particle size distribution of CQDs by enhancing the polymerization reaction. Notably, functional groups (-NH_2_, -CONH_2_, and -NO) can be used to adjust the emission wavelength of CQDs, thus achieving precise control of fluorescence performance. Similarly, mannose-functionalized CQDs could be used to specifically recognize *Escherichia coli*, while folate-functionalized CQDs had a good targeted recognition ability for tumor cells. In addition, the targeted binding efficiency and fluorescence stability of CQDs could be further optimized by regulating the ratio of mannan or folic acid to CQDs and the reaction temperature during the synthesis process. However, SMOCs do not possess the natural porous structure and ordered carbon skeleton of carbon-based materials, which lead to relatively low electrical conductivity of CQDs prepared from SMOCs as carbon sources (Yadav et al., 2023). Compared with biomass carbon sources, SMOCs have a more single composition, which makes them difficult to form a multi-doped carbon structure and limits the application performance of CQDs in fields such as electrocatalysis (Sikiru et al., 2023). In the future, studies on the preparation of CQDs using SMOCs as carbon sources should focus on precise structural regulation and functional synergistic optimization. Meanwhile, green synthetic pathways should be adopted to reduce energy consumption and by-product generation, further enhancing their application potential in energy conversion, environmental monitoring, and food packaging fields.

### 3.3 Biomass carbon sources

Biomass carbon sources refer to natural products or their wastes derived from animals and plants, such as fruit juice, plant leaves, straw, eggshell membranes, hair, discarded food, etc. (Troiano et al., 2023). Biomass is rich in components such as carbohydrates, proteins, and lipids. During the carbonization process, these components interact with each other and easily form multi-doped CQDs, which can endow CQDs with excellent fluorescence properties (Liu et al., 2020). Biomass molecules contain functional groups such as hydroxyl, amino, and carboxyl groups, which make the surface of the prepared CQDs rich in various functional groups. This is conducive to increasing the water solubility and biocompatibility of CQDs. Meanwhile, biomass carbon sources have the advantages of wide availability, low cost, and renewability, which are in line with the concept of green development (Li et al., 2022a, Li et al., 2022b, Li et al., 2022c). At present, increasing studies are focusing on the preparation of CQDs using resources such as agricultural waste, food residues, and biological waste. For instance, Preethi et al. (2022) prepared CQDs using rice as the carbon source. The results show that the prepared CQDs had an amorphous structure inside the nucleus, hydroxyl functional groups on the surface, and excellent water solubility. In addition, CQDs showed green fluorescence under ultraviolet lamp excitation, and CQDs had a highly selective response to Cu^2+^, which could be used for the fluorescence probe detection of Cu^2+^ in water samples. In another study, CQDs were prepared by the hydrothermal method using mangoes as the carbon source, and their structure and optical properties were investigated. The results indicate that there were oxygen and nitrogen-containing functional groups on the surface of CQDs, which were attributed to the carbonization and self-doping of sugars and proteins in mangoes during the hydrothermal process. In addition, the CQDs with a quantum yield of 8.39 % showed good water solubility and emitted blue fluorescence under 376 nm excitation, which could be used for Fe^3+^ detection and anti-counterfeiting labels (Ma et al., 2023). Wang et al. (2020) used biomass wastes such as orange peels, ginkgo leaves, paulownia leaves, and magnolia flowers as carbon sources to prepare CQDs by the hydrothermal method. The results show that the prepared CQDs were spherical, had a uniform particle size distribution, and possessed excellent fluorescence performance and stable optical properties. In addition, the surface of CQDs was rich in hydrophilic groups such as -OH, -COOH, and -NH_2_, which could endow them with good water solubility and significantly enhance their application potential in ion detection. However, compared with carbon-based materials and SMOCs, the preparation of CQDs from biomass as a carbon source has limitations, including poorer homogeneity, lower batch repeatability, and more difficult separation and purification. This is mainly attributed to the complex composition and natural heterogeneity of biomass, which makes it difficult to precisely control the reaction pathway during the carbonization process.

## 4 Antibacterial mechanisms of CQDs

CQDs, as nanomaterials with promising development prospects, have been confirmed by research to possess excellent antibacterial activity (Jeong et al., 2024). In addition, CQDs have gradually become the focus of materials science research due to their excellent biocompatibility, high hydrophilicity, rich surface functional groups, and low toxicity (Mahat et al., 2020). Numerous studies have confirmed that the main antibacterial mechanisms of CQDs include destroying the structure of bacteria, inducing the generation of ROS, disrupting biofilm, and improving antibiotic sensitivity (Wang et al., 2024; Xia et al., 2025a, Xia et al., 2025b; Liang et al., 2020). Notably, CQDs can reduce the development of bacterial resistance, indicating that CQDs can be used as efficient antibacterial agents for food preservation (Magagula et al., 2022). Currently, existing research mainly focuses on exploring the antibacterial properties of CQDs. However, the antibacterial mechanisms of CQDs remain unclear. Accordingly, this section systematically clarifies the antibacterial mechanisms of CQDs from different aspects.

### 4.1 Destroy the structure of bacteria

**Fig. 3:**
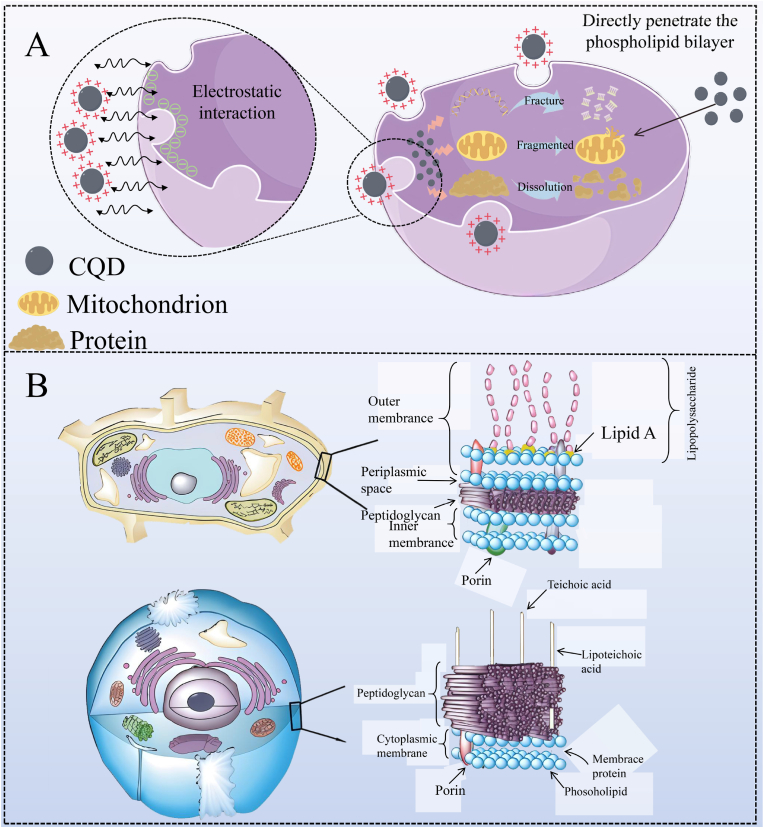
Mechanisms of CQDs destroying bacterial structure. A: Mechanisms of CQDs antibacterial through electrostatic action, directly penetrating thephospholipid bilayer, and destroying the membrane's integrity; B: The schematic diagram of the cell wall structure of *S. aureus* and *E. coli*. (Hao et al., 2021).

Presently, bacterial infections pose a significant threat to human safety. Besides, the excessive utilization of antibiotics can easily lead to bacterial resistance, which in turn can cause adverse reactions in clinical practice (Mao et al., 2018; Zhang et al., 2021a; Willyard, 2017). Hence, it is extremely important to prepare new, highly efficient, and safe antibacterial drugs and anti-drug-resistant bacteria. CQDs, as the new type of nanomaterials, have attracted much attention in the biomedical field, which is attributed to their excellent optical properties and water solubility, low toxicity, good biocompatibility, and abundant raw material resources (Iravani and Varma, 2020). Interestingly, CQDs can interact electrostatically with the bacterial membrane through surface charges, directly penetrate the phospholipid bilayer, and destroy the membrane's integrity, thus leading to bacterial death (Fig. 3A). For instance, Chai et al. (2022) found that the positive charge on the surface of CQDs could effectively inhibit the growth of Gram-negative and Gram-positive bacteria. Although CQDs could be adsorbed on the bacterial cells via electrostatic interaction, the loose but thick cell membrane structure of Gram-positive bacteria was more appropriate to absorb CQDs than that of Gram-negative bacteria with intensive and thin cell wall structure (Fig. 3B). In another study, the positively charged CQDs could adhere to the surface of bacteria through electrostatic action, destroying the integrity of the bacterial cell structure, thereby exerting antibacterial effects. Notably, the inhibitory effect of CQDs on Gram-positive bacteria was stronger than that on Gram-negative bacteria, which was mainly attributed to the strong adsorption capacity of CQDs on the loose and thick cell wall structure of Gram-positive bacteria (Hao et al., 2021). CQDs with positive charges showed a consistent minimum inhibitory concentration (MIC of 3 μg/mL) against both non-multidrug-resistant and multidrug-resistant strains. The antibacterial mechanisms of positively charged CQDs mainly involved strong electrostatic attraction and hydrophobic interaction between them and the negatively charged bacterial cell membranes, which caused damage to the bacterial cell membranes and inhibited bacterial growth, further suggesting that the positively charged CQDs have good antibacterial properties (Geng et al., 2021).

At present, existing research has confirmed that CQDs had the characteristic of ultra-small size, which could directly penetrate the lipid bilayer of bacteria, interact with DNA, proteins, and other substances within bacteria, affect bacterial metabolism, and promote bacterial apoptosis. Growing study focuses on taking advantage of the ultra-small size characteristics of CQDs to exert their antibacterial activity. Xu et al. (2024) prepared polylysine-derived CQDs (PL-CQDs) with an average particle size of 2.3 nm ± 0.36 nm, which could rapidly inactivate *Enterococcus faecali*s. The tiny size of PL-CQDs endowed them with a strong ability to penetrate the lipid bilayer of bacteria and promote interaction with DNA molecules in bacteria, thereby inducing bacterial apoptosis. In another study, the quaternary ammonium CQDs (QCQDs) with an average particle size of 5 nm could effectively penetrate the bacterial cell wall, act on the bacterial ribosome, interfering with the translation, modification, and turnover of proteins within the bacterial cells, thereby leading to metabolic disorders and death of the bacterial cells (Zhao et al., 2020).

In summary, CQDs are widely used in antibacterial materials due to their inherent physical properties (positive charge properties and nanoscale size) and good antibacterial properties. CQDs mainly exert their antibacterial activity through the following ways: 1) Destroying the structure of bacteria; 2) Disturbing cell membranes by strong electrostatic attraction and hydrophobic interaction; 3) affecting bacterial metabolism; 4) promoting the DNA, proteins, and other substances within bacteria. Regrettably, CQDs still face limitations in exerting their antibacterial effects, such as poor selectivity and stability, limited penetration barriers, susceptibility to environmental interference, and potential drug resistance. To the above limitations, we should optimize CQDs from the following aspects: 1) Ligands with bacterial specificity are attached to the surface of CQDs to enhance the enrichment of CQDs on the bacterial surface and reduce non-specific binding to non-target cells; 2) CQDs connect cell-penetrating peptides to overcome their structural barriers and biofilm matrix obstacles, and promote the exertion of antibacterial activity; 3) The development of CQDs sensitive to specific environmental factors is conducive to their activation at the target infection site, the release of antibacterial components, and the reduction of environmental interference caused by CQDs.

### 4.2 Induce the generation of ROS

**Fig. 4:**
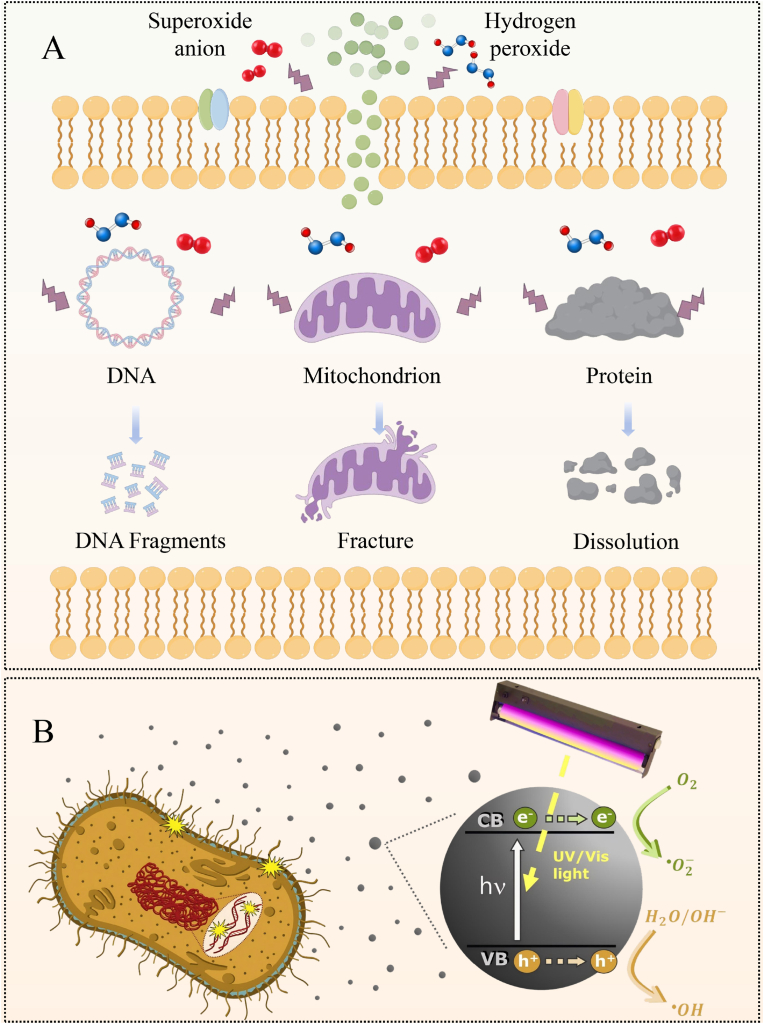
Antibacterial mechanisms diagram of CQDs. A: The bactericidal mechanisms of CQDs by inducing oxidative stress and damaging DNA, mitochondrion, and protein; B: In photocatalysis, light of energy greater than the band gap of a semiconductor excites an electron from the valence band (VB) to the conduction band (CB), creating a negatively charged free electron (e−) and a positively charged electron hole (h+). Electrons and holes may recombine, or, when they reach the surface, react with water and dissolved oxygen to give ROS, including ·O^2−^ and ·OH, resulting in antimicrobial effects (Parra-Ortiz & Malmsten, 2022).

Reactive oxygen species (ROS) are chemical substances containing highly reactive oxygen atoms, including OH·, ^1^O_2_, H_2_O_2_, etc. (Wang et al., 2021). ROS produced by the body's metabolism can be eliminated through protective mechanisms under physiological conditions (Wu et al., 2021a). However, excessive ROS can induce oxidative damage in cells, eventually leading to cell death (Li et al., 2022a, Li et al., 2022b, Li et al., 2022c). CQDs can enhance the destructive effect on bacteria by generating ROS, destroying the related structures of bacteria, and triggering the oxidative stress response of bacteria, thereby achieving the killing and inhibitory effects on bacteria (Fig. 4A). Interestingly, light is also a key factor in the generation of ROS in CQDs. This is mainly attributed to the fact that electrons around CQDs can transition from the valence band to the conduction band, generating electron-hole pairs that react with water and dissolved oxygen under light conditions, thus generating ROS (Fig. 4B) (Parra-Ortiz and Malmsten, 2022). Currently, numerous studies are applying CQDs to antibacterial materials by utilizing the ROS generation mechanism of CQDs. Wen et al. (2025) combined polyvinyl alcohol/chitosan films with CQDs to prepare wound dressings with antibacterial properties. The results indicate that the CQDs in the wound dressing could induce a large amount of ROS under light conditions (405 nm), thereby significantly inhibiting the growth of *Escherichia coli* and *Staphylococcus aureus*. In addition, the CQDs in the wound dressing could be adsorbed onto the negatively charged bacterial cell membrane through electrostatic action, destroying the membrane integrity, accelerating the leakage of contents, thereby demonstrating good antibacterial activity. In another study, turmeric was used as the carbon source to prepare CQDs with good photodynamic antibacterial activity via the solvent method. The results exhibit that CQDs produced a large amount of ROS under blue light irradiation, which could cause the cell membranes of *Escherichia coli* and *Staphylococcus aureus* to shrink, rupture, and lead to leakage of intracellular macromolecules, resulting in bacterial death (Yan et al., 2023). Ezati et al., 2022a, Ezati et al., 2022b, Ezati et al., 2022c prepared pectin/CQDs films by integrating CQDs into pectin films. The results show that the pectin/CQDs films could generate a large amount of ROS under light conditions, which could efficiently inactivate microorganisms, such as *Escherichia coli* and *Listeria*, thereby increasing their antibacterial activity. Besides, the pectin/CQDs films exhibited good biocompatibility and low cytotoxicity, further suggesting that the pectin/CQDs films show great application potential in the field of antibacterial food packaging. Similarly, S/P-CQDs with antibacterial activity were prepared by the hydrothermal method using aminophenol as the carbon source (Luo et al., 2023). The results show that the MIC values of S/P-CQDs against *Escherichia coli* and *Staphylococcus aureus* were 0.48 mg/mL. This is mainly attributed to the fact that S/P-CQDs induced bacteria to produce a large amount of ROS and promote the exudation of bacterial cytoplasm, thus causing bacterial apoptosis. Moreover, S/P-CQDs might penetrate into bacteria, disrupt the electron transfer process on mitochondria or membranes, and intensify oxidative stress, further suggesting that S/P-CQDs can be widely used as an antibacterial agent in the field of food preservation. In another study, N-CQDs were prepared by using the hydrothermal method and evaluated their antibacterial activity against drug-resistant bacteria. The results exhibit that N-CQDs could markedly reduce the bacterial survival rate of *Staphylococcus aureus* and methicillin-resistant *Staphylococcus aureus*. This was mainly attributed to the fact that N-CQDs could induce bacteria to produce a large amount of ROS under 525 nm light conditions, activate oxidative stress responses, damage bacterial structures, and ultimately lead to bacterial death (Xia et al., 2025a). Similarly, Li et al., 2020a, Li et al., 2020b prepared Lys-CQDs and Arg-CQDs by pyrolysis using lysine and arginine as raw materials. In addition, Lys-CQDs and Arg-CQDs could inhibit the activity of antioxidant enzymes in bacteria and generate a large amount of ROS, which in turn triggered lipid oxidation and membrane perforation. Furthermore, Lys-CQDs and Arg-CQDs were not prone to inducing bacterial resistance, further implying that Lys-CQDs and Arg-CQDs show great prospects in clinical antibacterial applications.

In summary, CQDs, as the new type of nanomaterial, can be excited under the condition of light irradiation. The activated CQDs can undergo photocatalytic reactions with water molecules and dissolved oxygen to produce ROS, disorder bacterial membrane structure, and inhibit physiological functions in bacteria, thereby achieving rapid and efficient broad-spectrum antibacterial effects. Unfortunately, the above-mentioned mechanisms face certain limitations in practical applications as follows: 1) Most CQDs that can efficiently generate ROS require external light sources for excitation, which limits their application in deep tissue infections or environments without light; 2) Excessive or untargeted ROS production inevitably causes oxidative damage to adjacent host cells, thereby leading to inflammatory responses or cell death; 3) CQDs have difficulty effectively entering host cells and locating to the location of bacteria. To address the above limitations, we can optimize them in the following methods: 1) Develop CQDs that can be stimulated by near-infrared light and enhance their penetration ability into tissues, thereby achieving deep therapeutic effects; 2) Prepare “intelligent” responsive CQDs that efficiently generate ROS only in the infected microenvironment, while minimizing damage to healthy tissues; 3) Modify cell-penetrating peptides or molecules that promote cell uptake on the surface of CQDs to enhance their efficacy against intracellular bacteria.

### 4.3 Disrupt biofilm

**Fig. 5:**
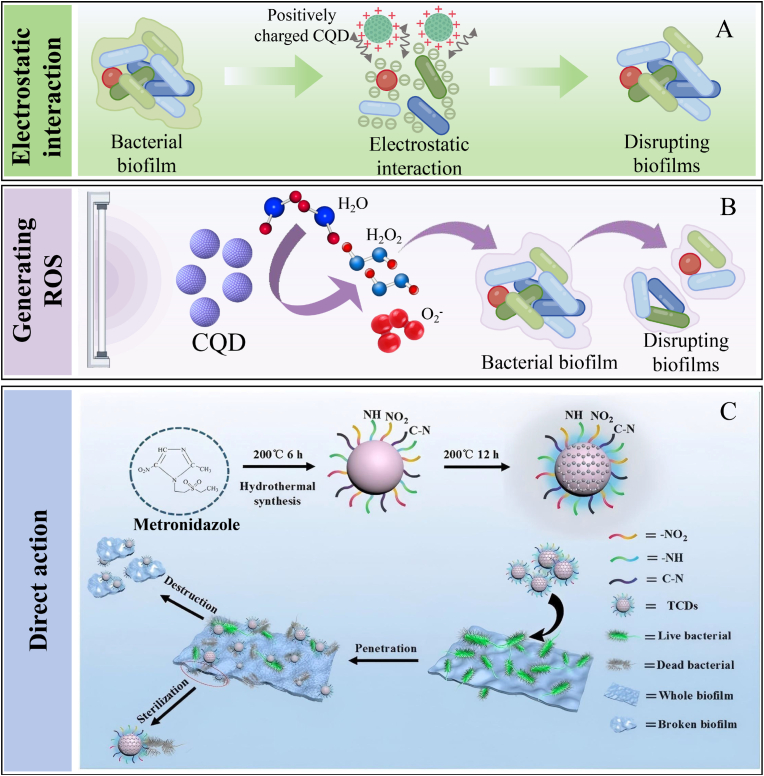
Diagram of the mechanism by which CQDs break down bacterial biofilms. A: CQDs break down the biofilm through electrostatic interaction; B: CQDs break down the biofilm by generating ROS; C: Schematic of specific anti-biofilm activity of CQDs (Liang et al., 2020).

Bacteria can adhere to the surfaces of bone/tooth structures/prosthesis implants during the infection process. Subsequently, bacteria can secrete extracellular polymers (EPS) on their surface, thereby forming biofilms (Wang et al., 2019d). Biofilms can provide a defense mechanism for bacteria and effectively resist the attack of the host immune system and the penetration of antibiotics (Davies, 2003). Destroying biofilms and inducing bacterial exposure to the immune system and antibiotics have become important action mechanisms of antibacterial for CQDs. Notably, CQDs can destroy polysaccharides, proteins, and other components in biofilms, induce the loosening of biofilm structures and the shedding of bacteria, thus enhancing the immune system and the killing effect of antibiotics on bacteria. For example, Wang et al. (2019b) synthesized N-CQDs using bisquaternary ammonium salts as carbon and nitrogen sources. The results exhibit that N-CQDs could kill the MRSA pathogen without causing drug resistance. In addition, N-CQDs could prevent the formation of biofilms and destroy established biofilms, thereby reducing the number of bacteria on infected tissues. Furthermore, N-CQDs could penetrate the loose network structure of EPS, directly act on the bacteria inside the membrane, and intensified oxidative stress, thereby enhancing the bactericidal effect. In another study, ethylenediamine-functionalized carbon quantum dots (EDA-CQDs) could interact with bacteria in the early stage of biofilm formation, thereby inhibiting bacterial adhesion and the initial deposition of EPS (Hindi et al., 2023). Besides, the functional groups on the surface of EDA-CQDs (-COOH, -OH, -NH_2_, etc.) could form hydrogen bonds or electrostatic interactions with polysaccharides and proteins in EPS, disrupt the stability of EPS, and cause the disintegration of the biofilm structure, thus exerting good antibacterial activity (Fig. 5A). Similarly, CQDs could destroy EPS in the biofilms of *Escherichia coli* and *Salmonella typhimurium* by generating excessive ROS (Fig. 5B). Moreover, excessive ROS could oxidize polysaccharides and proteins in EPS, disrupt the integrity of the biofilm, and directly attack the bacteria within the membrane, which could lead to lipid and protein peroxidation in bacteria, accelerate the degradation of quorum sensing (QS) signaling molecules in bacteria, thereby enhancing their antibacterial activity (Wu et al., 2021b). Wu et al. (2021a) synthesized CQDs by pyrolysis using carbon fibers as the carbon source. The results show that CQDs could specifically destroy the polysaccharide matrix in the biofilm of *Staphylococcus aureus* and reduce their protective barrier, which was conducive to the spread of pathogens and enhanced the sensitivity of pathogens to antibiotics. In addition, CQDs could competitively bind to the adhesins on the surface of *Staphylococcus aureus*, inhibit their adhesion to the material surface or host tissue, and prevent the formation of biofilms. Tinidazole CQDs (TCQDs) were prepared by the hydrothermal method using tinidazole as the carbon source (Liang et al., 2020). The results show that TCQDs could penetrate biofilms and effectively inhibit the growth of *Porphyromonas gingivalis*. In addition, TCQDs could affect the self-assembly of biofilm-related proteins by inhibiting the virulence factors and related genes of *Porphyromonas gingivalis* biofilm formation, which was conducive to increasing the killing effect of antibiotics on drug-resistant strains (Fig. 5C). In another study, polylysine-derived carbon quantum dots (PL-CQDs) were prepared through pyrolysis using lysine as the carbon source and evaluated their antibacterial activity. PL-CQDs could significantly reduce the amount of biofilm in the *in vitro* dental model, disrupt the compact structure of the biofilm, and enhance the sensitivity of bacteria to drugs. Besides, PL-CQDs could inhibit the QS system of bacteria, reduce the secretion of EPS, and decrease the maturity of biofilms. Furthermore, PL-CQDs possessed enzyme-like activity, which could catalyze the degradation of polysaccharide chains or proteins in EPS and weaken the mechanical strength of the coating, further suggesting that PL-CQDs show excellent antibacterial activity and great application potential in the biomedical field (Xu et al., 2024).

In summary, CQDs exert an anti-biofilm effect through the synergy of multiple molecular mechanisms as follows: 1) CQDs can generate electrostatic interaction with the bacterial cell membrane, disrupt the integrity of the bacterial membrane, and accelerate the leakage of contents, thus exhibiting better antibacterial activity; 2) CQDs can induce the production of ROS and effectively inhibit bacterial growth; 3) CQDs can competitively bind to adhesins on the surface of bacteria, inhibit bacterial adhesion, prevent the formation of biofilms, thereby exerting better antibacterial activity; 4) CQDs can accelerate the penetration into bacteria, degrade EPS, and promote bacterial apoptosis. However, the antibacterial activity of CQDs still faces limitations in practical applications, including limited penetration efficiency, susceptibility to interference from environmental factors, and easy induction of the emergence of new drug-resistant bacteria. In the future, we can take the following measures to optimize CQDs: 1) Introduce peptides with membrane penetration ability or ligands that can specifically recognize the components of biological membranes to promote the penetration of CQDs through the EPS barrier; 2) Enhance the stability of CQDs in complex environments through surface coating or cross-linking to reduce the interference of organic substances in the environment; 3) Reduce the risk of drug resistance development through combined treatment strategies.

### 4.4 Improve antibiotic sensitivity

**Fig. 6:**
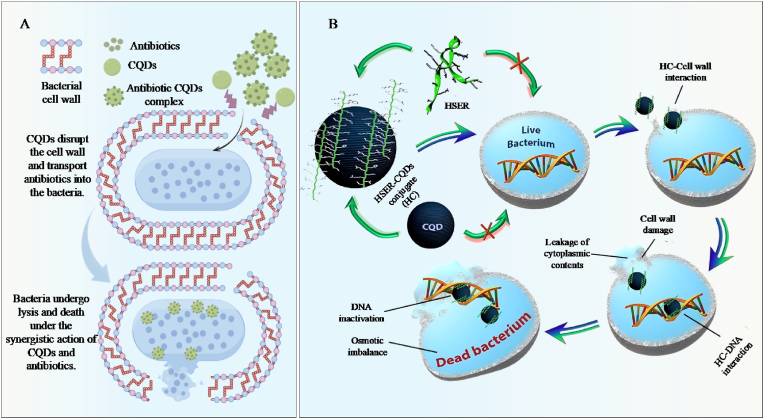
Diagram of the synergistic antibacterial mechanism between CQDs and antibiotics. A: CQDs disrupt the cell wall and transport antibiotics into the bacteria, and Bacteria undergo lysis and death under the synergistic action of CQDs and antibiotics; B: Peptide-CQDs conjugate against antibiotic-resistant Gram positive and Gram negative Pathogenic bacteria (Mazumdar et al., 2020).

The extensive utilization of antibiotics has led to the rapid emergence of drug-resistant strains, and single antibiotic treatment has become difficult to meet clinical needs (Xia et al., 2025a). Hence, how to enhance the efficacy of antibiotics and reduce drug resistance has become a research hotspot. CQDs possess the capability to directly kill bacteria and can also enhance the antibacterial efficacy of antibiotics through synergistic effects, thereby exerting antimicrobial activity (Zare et al., 2025). Interestingly, CQDs can enhance the antibacterial effect of antibiotics through multiple mechanisms. CQDs can target and transport antibiotics, destroy the cell walls and cell membranes of bacteria, which facilitates the entry of antibiotics into the interior of bacteria and increases the local concentration of antibiotics at the target site, thereby enhancing the sensitivity of antibiotics (Fig. 6A). For instance, Ardekani et al. (2019) prepared cCQDs via the hydrothermal method using chlorophyll as the carbon source, and then conjugated them with metronidazole (MET) for antibiotic drug delivery. The results indicate that compared with MET alone, the antibacterial activity of MET-cCQDs was more significantly increased by 72 %, which was mainly attributed to the fact that MET-cCQDs could deliver MET into cells, thereby increasing the concentration of MET within cells. Moreover, the combined antibacterial mechanisms of cCQDs and MET could significantly reduce the dosage of MET, while decreasing the emergence of drug-resistant bacteria. In another study, CQDs could destroy EPS in the biofilms of *Escherichia coli* and *Salmonella typhimurium* by generating a large amount of ROS and decreasing the bacterial volume density in the biofilms, which was conducive to increasing the contact space between antibiotics and bacteria, thereby enhancing the killing effect of antibiotics on bacteria (Wu et al., 2021b). Raut et al. (2023) prepared nitrogen-sulfur-doped CQDs (N,S-CQDs) by the microwave-assisted method using mercaptosuccinic acid and diethylenetriamine nitrogen as precursors. The results show that N,S-CQDs could exert a synergistic antibacterial effect with a variety of antibiotics (chloramphenicol, streptomycin amine, benzylpenicillin, etc.), disrupt the cell membrane structure, increase membrane permeability, and promote the entry of antibiotics into the cell. Numerous studies have confirmed that the combination of CQDs and antibiotics could significantly inhibit bacterial gene expression and kill antibiotic-free bacteria, thereby delaying the development of bacterial resistance, which was conducive to the continuous antibacterial activity of antibiotics (Zhang et al., 2018; Chatterjee et al., 2024). In addition, CQDs used in combination with antibiotics could interfere with the function of the efflux pump, reduce antibiotic efflux, and increase intracellular drug concentration, which was conducive to reducing bacterial resistance and enhancing the bactericidal effect of antibiotics. Fu et al. (2023) prepared WS-CQDs via the hydrothermal method using tryptophan and sorbitol as raw materials. The results indicate that WS-CQDs could directly damage bacterial DNA and inhibit the formation of bacterial biofilms. Besides, WS-CQDs could bind to drug-resistant genes (mecA and blaZ), inhibit their expression, and restore antibiotic sensitivity. In another study, the homo sapiens retinal receptor (HSER) peptide was combined with CQDs to prepare HSER-CQDs and evaluated their antibacterial effect on antibiotic-resistant bacteria (Mazumdar et al., 2020). The results show that HSER-CQDs could destroy the bacterial cell wall and interact with DNA, which led to DNA coagulation or conformational changes and affected their normal function (Fig. 6B). In addition, the sp^2^ carbon structure of HSER-CQDs could undergo π-π stacking with the bases of DNA (purines and pyrimidines), and then insert into the double helix structure of DNA, eventually causing local untwisting or breakage. Furthermore, HSER-CQDs could bind to the active centers of DNA polymerase or RNA polymerase, hinder their binding to DNA templates, and inhibit the DNA replication and gene transcription of bacteria. Li et al. (2023a) prepared composite films with antibacterial properties using chitosan, silver sulfide, and CQDs as raw materials. The results show that the inhibition rates of the composite films against *Escherichia coli* and *Staphylococcus aureus* were 86 % and 67 %, respectively, while the inhibition rate against drug-resistant bacteria was 73 %. Additionally, the dense structure of the composite film prevented bacterial adhesion and degraded EPS through Ag^+^ and ROS, thereby reducing the risk of drug resistance. Furthermore, the introduced CQDs could penetrate the biofilm and mechanically damage the bacterial membrane through the “nanoknife effect”, accelerating the penetration of intracellular substances and the death of bacteria.

In summary, the synergistic antibacterial effect of CQDs and antibiotics is mainly reflected in two aspects as follows: 1) CQDs can significantly enhance the sensitivity of bacteria to antibiotics; 2) CQDs can effectively reduce the formation of bacterial resistance, thereby significantly enhancing the antibacterial effect of traditional antibiotics. The dual-action mechanisms provide an innovative solution to the problem of drug-resistant bacterial infections. However, there are still certain limitations in the clinical practice of the synergistic antibacterial effect of CQDs and antibiotics as follows: 1) The molecular mechanisms of the synergistic antibacterial effect of CQDs and antibiotics are still unclear; 2) Research on the *in vivo* metabolic pathways of CQDs, potential organ toxicity due to long-term retention, and immunogenic reactions is still insufficient; 3) Surface modifications carry out to achieve targeting, enhance penetration or improve biocompatibility, and increase the complexity and cost of the production process. In the future, we should improve biocompatibility, reduce immunogenicity, and promote clearance in the body through precise surface modification. In addition, we can develop CQDs with naturally excellent properties and reduce the need for subsequent complex modifications.

## 5 The conditions of applying CQDs to food packaging

CQDs, as the new type of carbon-based nanomaterial, have attracted extensive attention in the field of food packaging due to their unique fluorescence properties, low cytotoxicity, and good biocompatibility (Chelladurai et al., 2024). Integrating CQDs into food packaging materials requires a comprehensive consideration of their dispersibility, stability, and compatibility with the matrix. This is conducive to maintaining the mechanical properties of raw materials and endowing packaging materials with new functional characteristics (Khan et al., 2023a, Khan et al., 2023b). Currently, physical mixing and chemical bonding are two important strategies for integrating CQDs into novel packaging materials. Among them, physical mixing involves directly dispersing CQDs into the polymer matrix. CQDs combine with the matrix material through van der Waals forces or hydrogen bonds, thereby endowing the matrix material with excellent antibacterial properties (Chaiwarit et al., 2025). For instance, Zheng et al. (2025) integrated CQDs into polyvinyl alcohol to prepare composite films and evaluated the effects of CQDs on the mechanical properties and antibacterial activity of the films. The results show that CQDs interacted with polyvinyl alcohol chains through hydrogen bonds, which increased the tensile strength and elongation at break of the composite film by 49.60 % and 49.55 % respectively. In addition, the film containing CQDs demonstrated significant inhibitory effects on *Escherichia coli* (the inhibition rate of 100 %) and *Staphylococcus aureus* (the inhibition rate of 89 %), which showed great application potential in the packaging of active foods. In another study, CQDs/pectin composite films were prepared by integrating CQDs into the voids of the pectin matrix through physical action, and the preservation effect of the composite films in the packaging of active foods was evaluated (Shen et al., 2024). The results show that CQDs could significantly enhance the mechanical and light barrier properties of the composite films, which was mainly attributed to the hydrogen bond interaction between CQDs and pectin molecules. In addition, CQDs could effectively enhance the cross-linking density between molecular chains, thereby restricting the free movement of polymer chain segments. Furthermore, the antibacterial activity of the CQDs/pectin composite films was positively correlated with the concentration of CQDs, indicating that an appropriate increase in the concentration of CQDs can significantly enhance the film's inhibitory ability against common food spoilage bacteria. Notably, the CQDs/pectin composite films could effectively delay the spoilage process of strawberries, significantly inhibit the growth of surface mold, and extend the shelf life by more than 5 days. Compared with chemical bonding, the physical mixing method is simpler, lower cost, less structural damage of CQDs, and better antibacterial activity (Ma et al., 2022). Unfortunately, physical mixing has limitations, including uneven dispersion of CQDs, easy agglomeration, and poor interfacial compatibility, which leads to a decline in the local performance of the material (Tran et al., 2025). Chemical bonding is achieved through the covalent connection between the functional groups on the surface of CQDs and the matrix material, which can enhance their stability in the matrix, prevent migration and agglomeration, and improve the overall performance of the composite materials (Zhu et al., 2024). For instance, Riahi et al. (2022) covalently combined CQDs with carboxymethyl cellulose to prepare the composite films with antibacterial properties and explored the preservation effect of the composite films on lemon fruits. The results show that CQDs covalently bond with the -CH_2_ groups on the carboxymethyl cellulose chain through -OH bonds, and CQDs were then uniformly distributed in the matrix. Carboxymethyl cellulose films had good compatibility with CQDs. In addition, the composite films exhibited excellent UV blocking properties, high light transmittance, and good water barrier performance. Furthermore, the composite films also showed excellent antioxidant, antibacterial, and antifungal activities, which could effectively inhibit the growth of microorganisms on the surface of lemon fruits and significantly extend their shelf life.

In summary, CQDs mainly combine with matrix materials through physical mixing and chemical bonding, thereby endowing packaging materials with functions such as antibacterial and antioxidant properties. In addition, integrating CQDs into food packaging can enhance the mechanical and barrier properties of packaging materials, thus improving their barrier capacity against external environmental factors such as oxygen and water vapor, and extending the shelf life of food. In the future, the performance of composite materials can be further optimized by regulating the size, surface functional groups, and interfacial interaction with the matrix of CQDs. With the development of smart packaging, food packaging materials integrated with CQDs can achieve real-time monitoring of food freshness and enhance the level of food safety management.

## 6 Applications of CQDs in food packaging

Food is prone to microbial infection in daily life, which in turn leads to spoilage. Therefore, food packaging can be used to inhibit bacterial growth, thereby extending the shelf life of food and maintaining food safety (Krepker et al., 2017). However, traditional packaging materials (such as polyethylene, polypropylene, polystyrene, etc.) have limitations, such as poor protection for food and difficulty in decomposition. Therefore, increasing studies are focusing on the development of new packaging materials. For example, bionic functional materials for bio-based food packaging have been proven to have significantly improved capabilities in all aspects compared to traditional food packaging materials and attract consumers with novel preservation functions (Zhong et al., 2025). Bionic technology can endow bio-based materials with excellent mechanical strength, optical properties, hydrophobicity, antibacterial properties, as well as novel responsiveness and other functions, which can also promote further progress and innovation in bio-based food packaging materials. In addition, Laccase (EC 1.10.3.2, various copper oxidase enzymes, globular proteins) is a class of enzymes with great potential in multiple industries and has received increasing attention in the field of food packaging (Sani et al., 2024). Laccase is used in food packaging films due to its biodegradability, ability to improve the quality and safety of packaged food, and reduce the need for chemical preservatives, which conform to consumers' preference for natural and healthy food. Laccase has biocompatibility and antibacterial properties, which can improve the physical, chemical, and functional properties of food packaging films by catalyzing phenolic polymerization, thereby enhancing mechanical strength, barrier performance, and thermal stability, Lactin can also be used to functionalize biopolymers through bioactive compounds such as antioxidants, antibacterial agents or flavorings to create packaging materials that not only protect food, but also provide additional health and sensory benefits. Integrating laccase into food packaging films is an important step towards sustainable packaging solutions. Notably, MOF, as a type of functional material, is particularly suitable for food safety due to its high surface area and porous structure (Khezerlou et al., 2025). MOF plays a crucial role in food packaging owing to its outstanding biocompatibility, antibacterial, and antioxidant properties, encapsulation capabilities, and non-reactivity when in contact with packaged products. Besides, MOF also has the unique ability to make colorimetric changes in response to environmental cues, which adds an innovative dimension to food packaging and provides consumers with visual indicators of product freshness and integrity. Interestingly, electrospinning, as an innovative multifunctional technology, can synthesize nanomaterials with unique properties and applications, thereby generating nanomaterials with a high surface area to volume ratio and enhancing mechanical properties and controllable porosity (Sree et al., 2025). Electrospun nanofibers loaded with active compounds (such as essential oils) can be incorporated into films to enhance their barrier properties against moisture, gases, and microorganisms, thereby extending the shelf life of perishable foods. Encapsulating antibacterial agents, antioxidants, and absorbents into nanofiber matrices can prevent food spoilage and foodborne diseases, thus ensuring the freshness and quality of agricultural products, which can effectively address issues related to plastic pollution and toxicity. Interestingly, increasing study is focusing on developing new types of nanocomposites to enhance the functionality and environmental friendliness of packaging materials. There are approximately 400 companies worldwide that focus on nanoparticles in food and food packaging (Perera et al., 2022). Research has found that there are currently relevant reports on integrating nanoparticles into the design and manufacturing processes of the packaging field. This novel approach combines the unique characteristics of nanoparticles (Singh et al., 2025). Currently, metal nanoparticles (silver nanoparticles, gold nanoparticles, copper nanoparticles, etc.) and metal oxide nanoparticles (zinc oxide, titanium dioxide, etc.) have advantages, including a broad antibacterial spectrum, strong antibacterial activity, and a low risk of developing drug resistance (Alavi et al., 2022). Hence, metal nanoparticles are applied in food packaging materials. Currently, Baby Dream Co. Ltd® (Korea) has produced a nano-silver baby bilk bottle, which was a baby bottle containing nano-silver and showed antibacterial properties, and Sharper Image® (U.S.) has produced a FresherLongerTM Miracle Food Storage, which was a food storage device using nano-silver (Al Mahmud, Mobarak and Hossain, 2024). Unfortunately, metal and metal oxide particles are prone to migrating into food and have high cytotoxicity, and long-term intake may pose potential risks to human health (Mortensen et al., 2020). In addition, the poor degradability and high cost of metal oxide nanoparticles greatly limit their application in the field of food packaging (Basavegowda et al., 2020). With the development of science and technology, CQDs show broad application potential in the field of food packaging due to their excellent antibacterial properties, superior biocompatibility, low cytotoxicity, and good antioxidant and anti-ultraviolet activities (Chelladurai et al., 2024). In the field of food packaging, bionic technology focuses on structural bionics. Electrospun nanofibers are good at loading active substances. MOFs are strong in adsorption and controlled release, and laccase is mainly used for biological sterilization. In contrast, CQDs possess highly efficient antibacterial and antioxidant properties, and can visually monitor the freshness of food through fluorescence changes. In addition, CDs are small in size and have high transparency, which does not affect the appearance of the packaging, and the raw materials of CDs have a wide range of sources, good biocompatibility, and they are safer and more environmentally friendly, which is conducive to achieving the integration of active preservation and intelligent indication. Hence, this section explores the application of CQDs in food packaging to reveal their unique advantages in inhibiting microbial growth and extending the shelf life of food. Table 2 exhibits the antibacterial effect of different CQDs and their effect on prolonging the shelf life of food.

**Table 2:** The preparation method, antibacterial effect, and preservation effect of CQDs.

Carbon source	Preparation method	Antibacterial effect	Shelf life	Reference
Waste ethanol	Hydrothermal method	The inhibitory effect on *Staphylococcus aureus* and *Escherichia coli* exceeds 85 % and 65 %, respectively.	Extend the shelf life of fruits by 8 d	Luan et al. (2025)
Cumin	Hydrothermal method	The diameters of the inhibition zones against *Listeria monocytogenes*, *Staphylococcus aureus*, *Enterococcus*, and *Escherichia coli* were 7.5 mm, 10.3 mm, 6.9 mm, and 3.1 mm, respectively.	Extend the shelf life of fruits by 24 d	Khan et al., 2024a, Khan et al., 2024b
Red capsicum annuum waste	Hydrothermal method	The diameters of the inhibitory zones against *Staphylococcus aureus*, *Enterococcus*, and *Escherichia coli* were 6.8 mm ± 0.50 mm, 4.5 mm ± 0.3 mm, and 3.9 mm ± 0.2 mm, respectively.	Extend the shelf life of fruits by 24 d	Riahi et al. (2025)
Amylaceum	Hydrothermal method	The bacterial count maintained at 6–7 log CFU/mL.	Extend the shelf life of fruits by 10 d	Ezati et al. (2022)
Amylaceum	Hydrothermal method	100 % effective against *Listeria monocytogenes* and *Escherichia coli*.	Extend the shelf life of fruits by more than 14 d	Ezati et al. (2022)
Radish peel	Hydrothermal method	The diameters of the inhibition zones against *Staphylococcus aureus* and *Escherichia coli* were 21 mm and 15 mm, respectively.	Extend the shelf life of meat by 8 d	Bahramian et al. (2025)
Coffee grounds	Hydrothermal method	The diameters of the inhibition zones for *Escherichia coli*, *Staphylococcus aureus*, and *Listeria monocytogenes* were 4.6 ± 0.3 mm, 6.3 ± 0.5 mm, and 12.7 ± 0.3 mm, respectively.	Extend the shelf life of meat by 21 d	Sul et al. (2024)
Mango peel	Hydrothermal method	The bacterial count was below 6 log CFU/g.	Extend the shelf life of meat by 15 d	Ponnusamy et al. (2025)
Cauliflora skins	Hydrothermal method	Reduce the growth of *Escherichia coli* by 8.1 log CFU/mL.	Extend the shelf life of seafood products by 15 d	Khan et al. (2024a)
*Citrus limon* acid and urea	Hydrothermal method	The counts of *Staphylococcus aureus* and *Listeria monocytogenes* decreased by 3.4 Log CFU/mL and 3.1 Log CFU/mL, respectively.	Extend the shelf life of seafood products by 18 d	Hong et al., 2025b
Yangmei fruit residue	Hydrothermal method	The inhibition rate against *Staphylococcus aureus* reached 89.95 %, and the inhibition rate against *Escherichia coli* was 94.60 %.	Extend the shelf life of seafood by 3 d	Yan et al. (2025)
Whey	Hydrothermal method	Maintain the bacterial count at 4 log CFU/g.	Extend the shelf life of cheese by 3–4 d	Lacivita et al. (2023)

### 6.1 Fruits and vegetables packaging

**Fig. 7:**
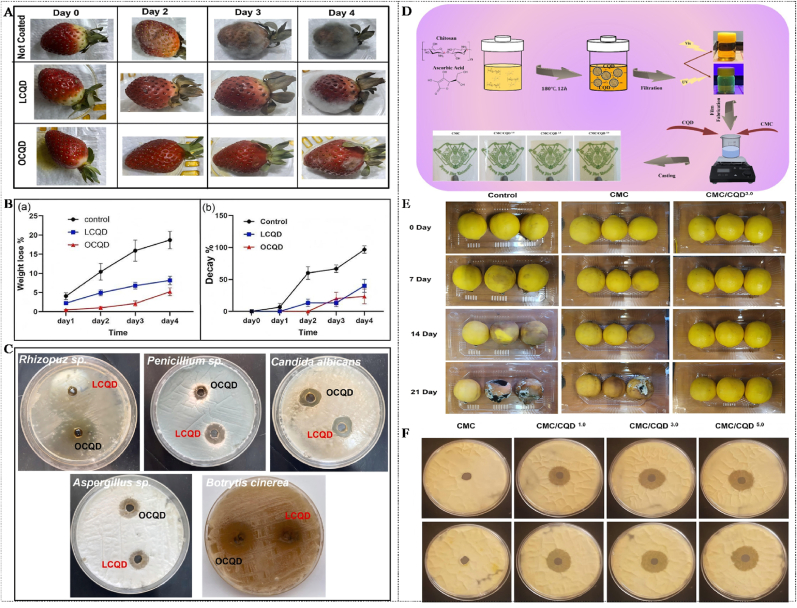
Application of food packaging film prepared based on CQDs in fruits and vegetables preservation. A: the excellent inhibitory effects on fungi in strawberries of LCQDs and OCQDs (Slewa, 2024); B: The effect of CQDs packaging film on extending the shelf life of strawberries (Slewa, 2024); C: The inhibition zone diameters for *Rhizopus sp., Penicillium sp., Candida albicans, Aspergillus sp.,* and *Botrytis cinerea* for LCQD and OCQDs (Slewa, 2024); D: The preparation of CMC/CQDs composite film (Riahi et al., 2022); E: The preservation effect of CQDs packaging film on lemons (Riahi et al., 2022); F: The size of the fungal growth inhibition zone of *A. niger* and *P. chrysogenum* strains (Riahi et al., 2022).

Fruits and vegetables, as living tissues, are highly susceptible to microbial damage after harvesting, which can lead to the occurrence of fruit and vegetable spoilage (Zhao et al., 2022). Among them, fungi are the main pathogens causing the spoilage of fruits and vegetables. For instance, *Botrytis cinerea* can cause tomato fruits to rot and produce harmful mycotoxins, threatening human health (Yang et al., 2023a, Yang et al., 2023b). *Penicillium* can cause penicillium disease in fruits such as citrus and apples, thus leading to fruit softening and rotting (Gandía et al., 2021). Therefore, taking effective measures to control the infection of fungal pathogens on fruits and vegetables is a key strategy to delay the spoilage process of fruits and vegetables. Notably, fungi have a strong cell wall structure and complex metabolic regulatory mechanisms, which make it difficult for traditional fungicides to effectively penetrate and exert a lasting bactericidal effect. Interestingly, CQDs can destroy the integrity of the fungal cell wall through dual mechanisms of physical perforation and oxidative stress, and effectively inhibit the infection and reproduction of fungi by interfering with bacterial metabolism and inhibiting mycelial growth and spore germination (Zhao et al., 2025). At present, increasing studies are focusing on exploring the application of CQDs in the field of fruit and vegetable packaging. In another study, lemon juice and onion juice were used as precursors to prepare lemon CQDs (LCQDs) and onion CQDs (OCQDs) by the hydrothermal method and explored their effect on strawberry preservation (Slewa, 2024). The results indicate that LCQDs and OCQDs exhibited excellent inhibitory effects on fungi in strawberries (Fig. 7A). Compared with the control group, strawberries coated with LCQDs and OCQDs on the surface had a longer shelf life (Fig. 7B). The calculated inhibition zone diameters for *Rhizopus sp., Penicillium sp., Candida albicans, Aspergillus sp.,* and *Botrytis cinerea* for LCQD were 44.5 ± 1.1, 18.9 ± 0.2, 18.5 ± 1.7, 15.2 ± 0.5, and 20.2 ± 1.3 mm, while for OCQD were 40.5 ± 2.5, 12.6 ± 0.52, 18.8 ± 2.08, 17.7 ± 2.5, and 26.16 ± 1.5 mm, respectively (Fig. 7C). Interestingly, the Zn-CQD/CGA complex was synthesized using chlorogenic acid (CGA) and Zn-CQDs as raw materials and explored its effect on the preservation of longan. The results indicate that the Zn-CQD/CGA complex could reduce the moldy, browning rate, and weight loss rate of longan peel and pulp. In addition, the Zn-CQD/CGA complex could adsorb bacterial membranes, disrupt their integrity, cause intracellular substance leakage, and inhibit microbial invasion of longan fruits, thereby extending the shelf life of longan (Du and He, 2024). Riahi et al. (2022) prepared CMC/CQDs composite films using CQDs and carboxymethyl cellulose (CMC) (Fig. 7D) and explored their preservative effect on lemons. The results exhibit that CMC/CQDs composite films could induce the generation of excessive ROS and attack the bacterial cell membrane, proteins, and DNA, thereby leading to bacterial death. Additionally, CQDs in the CMC/CQDs composite films could reduce the respiration intensity of lemons, decrease the consumption of nutrients (sugar and acid, the water vapor transmission rate, and water loss), thus extending their shelf life (Fig. 7E). Furthermore, the CQD-added CMC films exhibited strong antifungal activity against *A. niger* and *P. chrysogenum* strains, and the size of the fungal growth inhibition zone was directly proportional to the added CQD concentration (Fig. 7F). Similarly, CQDs was incorporated into guar gum (GG) and sodium alginate (SA) films to prepare GG/SA-CQDS composite films for asparagus preservation. The results indicate that CQDs in the composite films could destroy the cellular structure of microorganisms on the surface of asparagus by generating ROS, thereby inhibiting their growth and reproduction. In addition, the positive charge of CQDs could enhance the electrostatic interaction with the negatively charged microbial cell membranes and promote cell membrane rupture, thereby inhibiting microbial growth (Zhang et al., 2025).

In summary, CQDs can effectively inhibit the infection and reproduction of pathogenic bacteria on the surface of fruits and vegetables by physically penetrating, inducing the generation of ROS, destroying the cell wall structure, and interfering with the metabolism and reproduction of fungi. However, the packaging films of CQDs still have the following limitations in practical applications: 1) Strong environmental dependence and low antibacterial activity in dark environments; 2) Poor long-term stability and easy degradation; 3) Poor dispersibility and prone to agglomeration in the matrix, thereby affecting their functional properties. To address the above limitations, we can optimize CQDs through the following aspects: 1) Introduce photosensitive groups to enhance their ability to generate free radicals in the dark environment; 2) Enhance the environmental stability of CQDs through surface modification techniques, and delay their photodegradation and oxidative deactivation; 3) Improve the dispersion of CQDs in the polymer matrix and reduce the agglomeration phenomenon by covalent functionalization. In the future, with the continuous advancement of CQDs preparation technology and the gradual improvement of the safety assessment system, CQDs antibacterial films are expected to become intelligent, safe, and efficient packaging materials for fruits and vegetables preservation, which provides an important strategy for reducing food waste.

### 6.2 Meat packaging

**Fig. 8:**
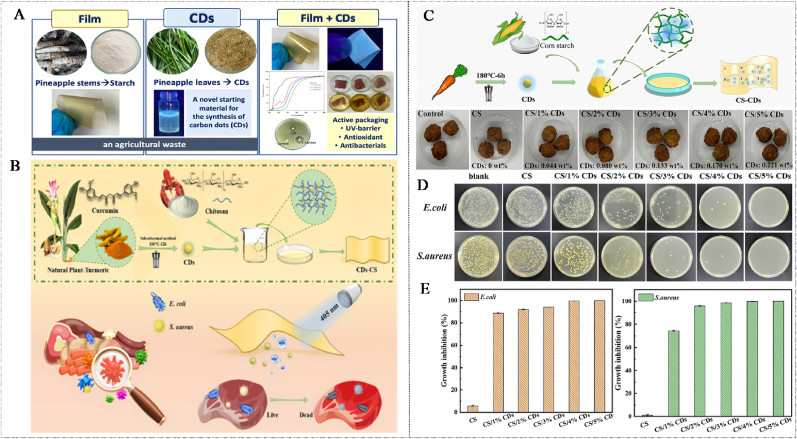
Application of CQDs antibacterial film in meat preservation. A: Preparation and application of PSS-CQDs film (Kuchaiyaphum et al., 2024); B: Structure and antibacterial strategy of the composite film (Wen et al., 2023); C: Preparation of the CQDs-CS composite films and extending the shelf life of meatballs (Wu et al., 2024); D and E: The antibacterial properties of the CQDs-CS composite film (Wu et al., 2024).

The spoilage of meat is mainly caused by its own enzymatic hydrolysis, oxidation reactions, as well as the growth and reproduction of microorganisms (Casaburi et al., 2015). Among them, the reproduction of microorganisms is the main factor causing meat spoilage. Especially, Gram-negative bacteria (such as *Pseudomonas* and *Escherichia coli*) and *Bacillus* can use protein and fat in meat as a source of nutrition, rapidly proliferate, and produce putrefactive substances (amines and sulfides), which can lead to darkening of meat color, softening of texture, and the generation of odors (Shao et al., 2021). Notably, bacteria form biofilms during the reproduction process, thus enhancing their tolerance to traditional antibacterial agents and accelerating the spoilage of meat (Mei et al., 2022; Sivakanthan et al., 2020; Zhang et al., 2021b). To ensure food safety, it is urgently necessary to develop new antibacterial technologies to reduce the risk of meat products being contaminated and extend their shelf life. Antibacterial packaging materials based on CQDs have shown broad application prospects in the field of food packaging due to their excellent antibacterial performance, good biocompatibility, and environmental friendliness (Chelladurai et al., 2024). CQDs are small in size and have a positive surface charge, which can electrostatically combine with EPS and penetrate bacterial biofilms, thus destroying the integrity of the membrane structure, which is conducive to increasing the sensitivity of bacteria to antibacterial agents, thereby synergistically enhancing the antibacterial effect (Li et al., 2020). In addition, CQDs can directly penetrate the bacterial cell membrane, induce the generation of ROS, cause intracellular oxidative stress, leakage of contents, and ultimately lead to bacterial death (Xia et al., 2025c). Notably, CQDs possess broad-spectrum antibacterial properties and can simultaneously inhibit multiple spoilage bacteria, thus effectively delaying the quality deterioration of meat during storage (Raul et al., 2022). Currently, increasing studies are focusing on the development of meat preservation materials by using CQDs antibacterial packaging films. For example, Kuchaiyaphum et al. (2024) doped CQDs into pineapple stem starch (PSS) to prepare a PSS-CQDs film (Fig. 8A) and explored its effect on the preservation of fresh pork. The results show that the positively charged groups on the surface of CQDs in the PSS-CQDs film could interact electrostatically with the negatively charged microbial cell membrane, destroy the integrity of the membrane, and accelerate the leakage of bacterial contents. In addition, CQDs in the PSS-CQDs film could inhibit the decomposition of myofibrin and connective tissue protein by secreted extracellular proteases (metalloproteinases, serine proteases, etc.) and reduce the production of free amino acids. Furthermore, the CQDs in the PSS-CQDs film could delay the change of color, odor, and other negative indicators of fresh pork, thereby extending the shelf life of fresh pork (Fig. 8A). Similarly, food packaging films with photodynamic sterilization properties were prepared by incorporating CQDs into chitosan matrix (Fig. 8B). The results show that CQDs in films could effectively delay the spoilage process of pork by destroying the adhesion structures such as microbial flagella and fimbriae, reducing their adhesion rate on the meat surface and the secretion activities of protease and lipase. In addition, the CQDs in films could enter the microbial cells, interfere with the activities of enzymes related to the electron transport chain and energy metabolism, and bind to DNA to affect the replication of genetic material, and cause the growth of microorganisms to stagnate, thus significantly inhibiting the spoilage of meat, further suggesting that the films show great application potential in the field of food packaging (Wen et al., 2023). In another study, CQDs were introduced into corn starch (CS) matrix to prepare CQDs-CS composite films (Fig. 8C) and evaluated their fresh-keeping effect on fried meatballs (Wu et al., 2024). The CQDs-CS composite films could maintain the appearance, smell, and quality of meatballs, thereby extending the shelf life of meatballs (Fig. 8C). Besides, CQDs in the CQDs-CS composite film could inhibit the growth of *E. coli* and *S. aureus* (Fig. 8D and E), destroy the integrity of the bacterial membrane, thus leading to the leakage of intracellular dissolved matter, further suggesting that the CQDS-CS composite films show a good fresh-keeping effect on the meatballs. Similarly, NP-CQDs were incorporated into chitosan/starch (Chi/St) to prepare active packaging films and explored their preservation effects in meat products. The results show that NP-CQDs in the films could block the initial colonization of bacteria, inhibit the transmission of signal molecules such as N-acyl hyperserine lactone (AHLs), reduce bacterial energy metabolism, and effectively reduce the growth of bacteria on the surface of meat products, which was conducive to extending the shelf life of meat products. Furthermore, the incorporation of NP-CQDs could undermine the resistance of bacteria to antibiotics and enhance the inhibitory effect of antibiotics on drug-resistant bacteria, thereby achieving synergistic antibacterial effects in composite applications (Khan et al., 2023).

In summary, CQDs can exert antibacterial effects through multiple mechanisms, including disrupting microbial structure, enhancing the sensitivity of spoilage bacteria to antibacterial agents, and inhibiting the formation of biofilms, thereby effectively delaying the spoilage process of meat. In addition, the introduction of CQDs not only enhances the antibacterial performance of packaging materials, but also endows them with functions such as anti-oxidation and UV blocking, further improving the comprehensive performance of food packaging materials. However, CQDs packaging films still have the following limitations in practical application: 1) The potential impact of long-term intake of CQDs on intestinal flora and organs lacks systematic assessment; 2) Compared with traditional antibacterial agents, the preparation cost of CQDs is higher, which affects their commercialization. 3) Some CQDs rely on contact for sterilization and lack a long-lasting release mechanism, resulting in a short duration of antibacterial activity. In view of the above limitations, we should focus on the following aspects to optimize CQDs: 1) Design CQDs immobilization technology to ensure that it is not released into food, thereby reducing the intake of CQDs; 2) Optimize the preparation process of CQDs, utilize biomass waste as the carbon source, and develop continuous flow reactors to reduce the preparation cost of CQDs; 3) Load CQDs onto porous materials to achieve controllable release. With the continuous improvement of CQDs preparation technology and safety assessment systems, CQDs antibacterial packaging will gradually move from the laboratory to large-scale applications, which can provide a safer and more efficient solution for food preservation in the future.

### 6.3 Seafood packaging

**Fig. 9:**
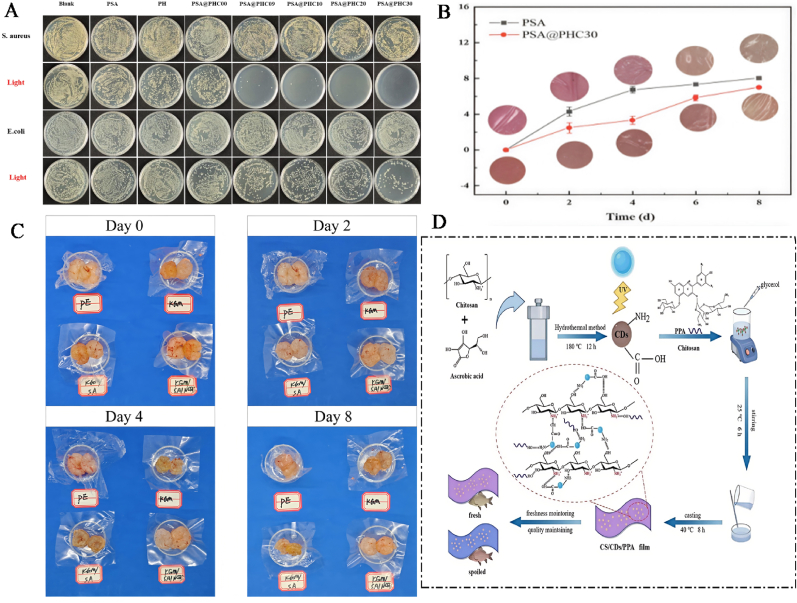
Application of CQDs packaging film in seafood. A: Inhibitory effect of novel anthocyanins film @carbon quantum dot nanofiber intelligent active double-layer film on bacteria (Qin et al., 2024); B: Images of crayfish meat packaged with different composite films during storage at 4 °C (Qin et al., 2024); C: The extending the shelf life of crayfish meat of N-CQDs in the films (Jiang et al., 2025); D: The synthesis of CDs and CS/PPA/CDs composite films (Hu et al., 2025).

Microbial contamination is a key factor leading to the deterioration of seafood quality, which is attributed to the fact that seafood is rich in high protein, unsaturated fatty acids, and high water activity (Tavakoli et al., 2022). Among them, *Psychrophilic bacteria* (*Pseudomonas*, *Shiva, Vibrio parahaemolyticus*, etc.) are the main bacterial groups causing the spoilage of seafood. These bacteria can secrete extracellular enzymes (protease and lipase), decompose proteins and fats, and produce substances with fishy odors and spoilage effects, including TMA, H_2_S, and free fatty acids, thereby leading to the deterioration of seafood (Ge et al., 2017). Therefore, inhibiting the growth of microorganisms in seafood has become a key link in the preservation of seafood. Traditional packaging materials often acquire antibacterial activity by adding metals or metal oxides, which not only cause environmental pollution, but also pose a threat to human health (Wei et al., 2025). Compared with traditional antibacterial agents, the low-temperature antibacterial activity of CQDs was more prominent, which could effectively inhibit the metabolism and reproduction of psychrophilic bacteria under refrigeration conditions. This was mainly attributed to the fact that CQDs were not dependent on temperature and could produce ROS under photocatalytic action, destroy the cell membrane structure of psychrophilic bacteria, and interfere with their enzyme functions, thereby inhibiting bacterial proliferation and achieving a low-temperature antibacterial effect (Wang et al., 2025a, Wang et al., 2025b). At present, increasing study is focusing on the utilization of CQDs antibacterial packaging materials to extend the shelf life of seafood. Qin et al. (2024) prepared composite films using CQDs and anthocyanins and explored their effect on fish meat preservation. The results show that CQDs in the films could attack the membrane lipids, proteins, and DNA of bacteria by generating ROS, which could cause oxidative damage and induce bacterial death, thereby demonstrating a good preservation effect on fish meat. Moreover, CQDs in the films bound to the negatively charged bacterial cell membranes (such as *Escherichia coli* and *Staphylococcus aureus*) through electrostatic adsorption (Fig. 9A), disrupting the order of the lipid bilayer of the cell membrane, increasing permeability, and causing the leakage of intracellular substances (such as ATP and alkaline phosphatase), which were conducive to extending the shelf life of fish meat (Fig. 9B). Similarly, biodegradable antibacterial food packaging films were prepared using N-CQDs, konjac glucomannan (KGM), and sodium alginate (SA) as raw materials and evaluated their preservation effects on crayfish meat (Jiang et al., 2025). The results show that N-CQDs in the films could continuously produce ROS under light, effectively destroy the cellular structure of spoilage bacteria in crayfish, and inhibit their growth and reproduction. In addition, the N-CQDs in the films could penetrate the EPS of bacterial biofilms, disrupt the structure of mature biofilms, reduce the number of live bacteria, thereby extending the shelf life of crayfish meat (Fig. 9C) . In another study, packaging materials with antibacterial properties were prepared by coating CQDs onto bamboo fiber paper substrates (Li et al., 2023b). The CQDs in the packaging materials could significantly reduce the number of bacteria on the surface of fresh shrimp, accelerate the destruction of bacterial cell membrane integrity, inhibit protein decomposition and fat oxidation, and delay the decay rate of fresh shrimp, further suggesting that the packaging materials have a wide range of application prospects in seafood preservation. Similarly, functional composite films for fish preservation were prepared by combining CQDs with chitosan (CS) and purple sweet potato anthocyanin (PPA) (Fig. 9D). The results indicate that the CQDs in the composite films were small in size and rich in positive charges, which facilitated their interaction with negatively charged bacterial cell membranes, enhanced membrane surface adsorption capacity, improved the contact efficiency between CQDs and bacteria, enhanced their ability to penetrate cell membranes, thus leading to membrane potential imbalance, ion leakage, and cell lysis. In addition, the continuous production of ROS by CQDs under light could synergistically disrupt the intracellular antioxidant system, exacerbate oxidative stress, effectively inhibit the growth of common spoilage and pathogenic bacteria in various aquatic products, and greatly improve the preservation performance (Hu et al., 2025).

In summary, CQDs composite films can effectively inhibit the growth of microorganisms in seafood, reduce the generation of putrefaction gases, thereby extending the shelf life of the products, which can provide an innovative solution for seafood packaging. Regrettably, CQDs composite films still have certain limitations in the field of seafood preservation as follows: 1) CQDs is prone to agglomeration in the polymer matrix, which affects the uniformity, transparency and performance of the film; 2) CQDs can interact with endogenous components in seafood and affect the biological activities and stability of CQDs; 3) CQDs may slowly release from the film or leach into the surface or juice of seafood, thus causing safety issues. To address the above limitations, we should focus on optimizing CQDs as follows: 1) Implement surface functionalization modification to enhance the dispersion and interfacial compatibility of CQDs in the matrix; 2) Combine CQDs with natural antibacterial agents to enhance the antibacterial performance of the material; 3) Fix CQDs in the polymer network or carrier through chemical/physical bonds embedding to reduce release/leaching. In the future, with the continuous advancement of CQDs preparation technology, CQDs composite films are expected to break through the current bottlenecks of preservation technology and further enhance the preservation effect and safety of seafood.

### 6.4 Other food packaging

Grains and dairy products, as important components of the human diet, contain a variety of nutrients and high water content, which can provide favorable conditions for the growth and reproduction of microorganisms. The rapid growth of microorganisms on grains and dairy products can easily lead to contamination by spoilage bacteria, generating harmful substances such as toxins, which pose a serious threat to food safety and consumer health (Fox et al., 2017; Fleurat-Lessard, 2017). The spoilage of grains (wheat, rice, and corn) and their processed products (flour, bread, and pastries) is mainly caused by mold. Molds can secrete extracellular enzymes such as amylase and cell wall hydrolase, which degrade the polysaccharide structure in grains and damage the cell wall structure of grains, thereby causing grains to mold. In addition, molds can secrete carcinogenic and teratogenic mycotoxins (aflatoxin, ochratoxin, and zearalenone). Mycotoxins are highly stable and difficult to remove through conventional processing, which seriously threatens human health (Khalil et al., 2021). Dairy products are susceptible to microbial contamination, including *Lactic acid bacteria*, *Pseudomonas, Bacillus*, mold, and yeast, which can lead to lactose fermentation, protein breakdown, and fat hydrolysis in dairy products, resulting in spoilage such as rancidity and clumping (Wang et al., 2025a, Wang et al., 2025b). Therefore, the inhibition of spoilage bacteria growth by antibacterial agents is the key to ensuring the quality and safety of grains and dairy products. However, the chitin structure in the cell walls of molds is dense, which makes it difficult for conventional antibacterial agents to penetrate effectively. In addition, there are numerous types of microorganisms causing spoilage in dairy products, and traditional antibacterial agents are difficult to achieve broad-spectrum antibacterial effects (Elbehiry et al., 2025). Notably, the surface of CQDs is rich in positive charges, which can act on the negatively charged fungal cell walls through electrostatic adsorption, thereby destroying their integrity. In addition, CQDs can generate ROS under light, destroy the cell membranes, proteins, and DNA of bacteria, thereby achieving efficient inhibition of molds and various spoilage bacteria (Tiras and Onmaz, 2025). Therefore, the application of CQDs in packaging materials for grains and dairy products to extend the shelf life of food and ensure safety is becoming a research hotspot. For instance, Lacivita et al. (2023) used whey as the carbon source to prepare an active packaging film containing CQDs by the hydrothermal method and explored its preservation effect on cheese. The results show that the film could significantly inhibit the growth of *Pseudomonas* and *Enterobacter* in cheese, thereby extending the shelf life of cheese and maintaining its sensory quality. This was attributed to the fact that CQDs could adsorb onto the surface of *Pseudomonas* through electrostatic interaction, generating ROS, disrupting the cell membrane structure, and inhibiting the growth of *Pseudomonas*. In addition, CQDs could also induce oxidative stress in *Pseudomonas*, disrupt the metabolic system, and inhibit the reproduction of *Pseudomonas*. In another study, CQDs were compounded with chitosan to prepare a film for extending the shelf life of bread. The results show that CQDs were small in size and evenly dispersed, and could effectively penetrate the bacterial cell wall. In addition, the ROS produced by CQDs under light exposure could disrupt the membrane structure and metabolic function of microorganisms, and significantly inhibit the reproduction of molds and putrefactive bacteria. In addition, the synergistic effect of CQDs and chitosan could enhance the mechanical properties of the film, improve the physical strength and barrier performance of packaging materials, and further delay the deterioration of food quality (Khan et al., 2025).

In summary, CQDS-based composite packaging materials can effectively inhibit the growth of mold and the production of mycotoxins in grains, delay the reproduction of various spoilage bacteria in dairy products, thereby significantly extending the shelf life of food. However, CQDs still face the following limitations in practical applications: 1) The release rate of CQDs in packaging materials is difficult to control, which may lead to a short duration of antibacterial activity in packaging materials; 2) CQDs in discarded packaging may pose potential hazards to environmental microorganisms; 3) The antibacterial stability of CQDs in complex food matrices is easily affected by environmental factors. To address the above limitations, we should optimize the following aspects: 1) Optimize the loading mode and embedding technology of CQDs to control their release rate; 2) Develop degradable carrier materials to reduce the potential environmental risks of CQDs; 3) Enhance the stability of CQDs under different conditions through surface functionalization modification. In the future, the application of CQDs in the packaging of grains and dairy products will develop in the direction of intelligence, precision, and sustainability, which can further ensure food safety and reduce food waste.

## 7 Conclusions and future prospects

CQDs, as the new type of nanomaterials, have attracted much attention due to their efficient antibacterial properties, unique optical properties, good biocompatibility, low cytotoxicity, and environmental friendliness. In recent years, great progress has been made in the preparation method, antibacterial mechanisms, and application of CQDs in the field of food packaging. Firstly, this paper reviews the preparation methods (arc discharge, laser etching, electrochemical exfoliation, etc.) of CQDs and the selection of carbon sources (carbon-based materials, small molecule organic compounds, and biomass carbon sources). Subsequently, this review systematically summarizes the antibacterial mechanisms of CQDs. Finally, this article comprehensively outlines the application of CQDs in the packaging of fruits and vegetables, meats, seafood, grains, and dairy products, which provide new ideas and methods for solving food safety problems and reducing food waste.

CQDs have achieved remarkable results in laboratory research and initial applications. Regrettably, CQDs still have many limitations in practical promotion and large-scale application as follows: 1) Due to the diversity and complexity of CQDs preparation methods, there are significant differences in CQDs in terms of particle size distribution, surface functional groups, and yield. Moreover, there is a lack of unified standards and norms, which greatly limits their application in different fields; 2) The long-term stability, migration behavior of CQDs in food contact materials, and their action mechanisms in complex food matrices still lack systematic research. Food packaging not only needs to ensure short-term antibacterial effects, but also needs to withstand the tests of complex environmental factors (temperature, humidity, and pH). The functional retention and safety of CQDs under these conditions still need further in-depth research; 3) The safety assessment of long-term exposure to CQDs is still insufficient, especially the possible metabolic pathways and potential cumulative effects after human intake have not been fully clarified; 4) CQDs still faces problems such as low yield, difficult purification, and high cost during the preparation process, which limit their application in different fields. To solve the above problems, future research should be improved in the following directions: 1) We need to establish a standardized CQDs preparation and characterization system to form unified quality evaluation indicators, which provide a solid foundation for the large-scale preparation and application of CQDs; 2) We should enhance interdisciplinary research to deeply reveal the mechanisms of CQDs in the food packaging environment and their interaction rules with food components; 3) We should develop efficient, low-cost, and environmentally friendly large-scale preparation technologies to obtain high-performance and special-function CQDs, which provides an important strategy for meeting the demands of industrial applications. In summary, CQDs, with their unique physicochemical properties and antibacterial functions, have injected new vitality into the food packaging field. With the optimization of the synthesis process and the in-depth study of the antibacterial mechanisms, this will be conducive to revealing the safety and functional characteristics of CQDs *in vivo* and *in vitro* in the future. In addition, CQDs are expected to achieve breakthroughs in green synthesis, precise regulation, safe application, and intelligent development, which is conducive to promoting the transformation of food packaging from traditional protective functions to efficient, intelligent, and sustainable development.

## CRediT authorship contribution statement

Hongkun Xue, Jiacheng Yang, and Hao Yu: Conceptualization, Formal analysis, Supervision, Data curation, Writing-original draft. Rong Dong: Visualization and Project administration. Xiaojun Liao and Jiaqi Tan: Funding acquisition, Project administration, Writing-review & editing.

## Declaration of competing interest

The authors declare that they have no known competing financial interests or personal relationships that could have appeared to influence the work reported in this paper.

## Data Availability

Data availability is not applicable to this article as no new data were created or analyzed in this study.
